# International Society of Sports Nutrition Position Stand: Probiotics

**DOI:** 10.1186/s12970-019-0329-0

**Published:** 2019-12-21

**Authors:** Ralf Jäger, Alex E. Mohr, Katie C. Carpenter, Chad M. Kerksick, Martin Purpura, Adel Moussa, Jeremy R. Townsend, Manfred Lamprecht, Nicholas P. West, Katherine Black, Michael Gleeson, David B. Pyne, Shawn D. Wells, Shawn M. Arent, Abbie E. Smith-Ryan, Richard B. Kreider, Bill I. Campbell, Laurent Bannock, Jonathan Scheiman, Craig J. Wissent, Marco Pane, Douglas S. Kalman, Jamie N. Pugh, Jessica A. ter Haar, Jose Antonio

**Affiliations:** 1 Increnovo LLC, Milwaukee, WI USA; 2 College of Health Solutions, Arizona State University, Phoenix, AZ USA; 3 Isagenix International LLC, Gilbert, AZ USA; 4 Exercise and Performance Nutrition Laboratory, School of Health Sciences, Lindenwood University, St. Charles, MO USA; 5 University of Münster, Department of Physics Education, Münster, Germany; 6 Exercise and Nutrition Science Graduate Program, Lipscomb University, Nashville, TN USA; 7 Otto Loewi Research Center, Medical University of Graz, Graz, Austria; 8 School of Medical Science and Menzies Health Institute of QLD, Griffith Health, Griffith University, Southport, Australia; 9 Department of Human Nutrition, University of Otago, Dunedin, New Zealand; 10 School of Sport, Exercise and Health Sciences, Loughborough University, Loughborough, UK; 11 Research Institute for Sport and Exercise, University of Canberra, Canberra, ACT 2617 Australia; 12 WGI, Lewisville, TX USA; 13 UofSC Sport Science Lab, Department of Exercise Science, University of South Carolina, Columbia, SC USA; 14 Applied Physiology Laboratory, Department of Exercise and Sport Science, University of North Carolina, Chapel Hill, NC USA; 15 Exercise & Sport Nutrition Lab, Human Clinical Research Facility, Department of Health & Kinesiology, Texas A&M University, College Station, TX USA; 16 Performance & Physique Enhancement Laboratory, University of South Florida, Tampa, FL USA; 17 Institute of Performance Nutrition, London, UK; 18 Fitbiomics, Inc, New York, NY USA; 19 Jamieson Wellness Inc, Windsor, Ontario Canada; 20 Bioloab Research, Novara, Italy; 21 Scientific Affairs. Nutrasource Diagnostics, Inc. Guelph, Guelph, Ontario Canada; 22 Research Institute for Sport and Exercise Sciences, Liverpool John Moores University, Tom Reilly Building, Byrom St Campus, Liverpool, UK; 23 International Probiotic Association, Los Angeles, CA USA; 24 Exercise and Sport Science, Nova Southeastern University, Davie, FL USA

**Keywords:** Gut-muscle-Axis, Microbiome, Microbiota, Sport performance, Muscle

## Abstract

**Position statement:** The International Society of Sports Nutrition (ISSN) provides an objective and critical review of the mechanisms and use of probiotic supplementation to optimize the health, performance, and recovery of athletes. Based on the current available literature, the conclusions of the ISSN are as follows:

1. Probiotics are live microorganisms that, when administered in adequate amounts, confer a health benefit on the host (FAO/WHO).
2. Probiotic administration has been linked to a multitude of health benefits, with gut and immune health being the most researched applications.
3. Despite the existence of shared, core mechanisms for probiotic function, health benefits of probiotics are strain- and dose-dependent.
4. Athletes have varying gut microbiota compositions that appear to reflect the activity level of the host in comparison to sedentary people, with the differences linked primarily to the volume of exercise and amount of protein consumption. Whether differences in gut microbiota composition affect probiotic efficacy is unknown.
5. The main function of the gut is to digest food and absorb nutrients. In athletic populations, certain probiotics strains can increase absorption of key nutrients such as amino acids from protein, and affect the pharmacology and physiological properties of multiple food components.
6. Immune depression in athletes worsens with excessive training load, psychological stress, disturbed sleep, and environmental extremes, all of which can contribute to an increased risk of respiratory tract infections. In certain situations, including exposure to crowds, foreign travel and poor hygiene at home, and training or competition venues, athletes’ exposure to pathogens may be elevated leading to increased rates of infections. Approximately 70% of the immune system is located in the gut and probiotic supplementation has been shown to promote a healthy immune response. In an athletic population, specific probiotic strains can reduce the number of episodes, severity and duration of upper respiratory tract infections.
7. Intense, prolonged exercise, especially in the heat, has been shown to increase gut permeability which potentially can result in systemic toxemia. Specific probiotic strains can improve the integrity of the gut-barrier function in athletes.
8. Administration of selected anti-inflammatory probiotic strains have been linked to improved recovery from muscle-damaging exercise.
9. The minimal effective dose and method of administration (potency per serving, single vs. split dose, delivery form) of a specific probiotic strain depends on validation studies for this particular strain. Products that contain probiotics must include the genus, species, and strain of each live microorganism on its label as well as the total estimated quantity of each probiotic strain at the end of the product’s shelf life, as measured by colony forming units (CFU) or live cells.
10. Preclinical and early human research has shown potential probiotic benefits relevant to an athletic population that include improved body composition and lean body mass, normalizing age-related declines in testosterone levels, reductions in cortisol levels indicating improved responses to a physical or mental stressor, reduction of exercise-induced lactate, and increased neurotransmitter synthesis, cognition and mood. However, these potential benefits require validation in more rigorous human studies and in an athletic population.

## Introduction

The term probiotic is derived from the Latin preposition “pro,” which means “for” and the Greek word “biotic” meaning “life”. Probiotics are widely considered to be health-promoting microorganisms. As outlined in Table 1 and as defined by the World Gastroenterology Organization (WGO), various ingredients can function in probiotic, prebiotic, and symbiotic roles. The Food and Agriculture Organization of the United Nations (FAO) and the World Health Organization (WHO) defines probiotics as “live microorganisms that, when administered in adequate amounts, confer a health benefit on the host” [1]. Additionally, the International Olympic Committee (IOC) has stated that, “Probiotics are live micro-organisms that when administered orally for several weeks can increase the numbers of beneficial bacteria in the gut. These have been associated with a range of potential benefits to gut health, as well as modulation of immune function” [5]. Unique in comparison to other dietary supplements, probiotic preparations contain live, viable, defined microorganisms in sufficient numbers to provide beneficial health effects [6]. Table 1 provides an overview of common definitions and classifications related to probiotic research.

**Table 1:** Definitions of common terminology and classifications in probiotic research

Concept	Definition
Probiotics	Live microorganisms which, when administered in adequate amounts, confer a health benefit on the host [1].
Prebiotic	A substrate that is selectively utilized by host microorganisms conferring a health benefit on the host [2].
Synbiotics	A synbiotic product beneficially affects the host in improving the survival and implantation of live microbial dietary supplements in the gastrointestinal tract by selectively stimulating the growth and/or activating the metabolism of one or a limited number of health-promoting bacteria [3].
Postbiotics	Postbiotics are bioactive components produced by beneficial bacteria (through a natural fermentation process) which have biological activity in the gut (e.g. short-chain fatty acids) [4].
Immunobiotics	Inactivated probiotics (e.g. heat-killed), in which the dead cells maintain their immune benefit.
Gut	The gastrointestinal tract is a long tube that starts in the mouth and ends at the anus. Its main function is to process food. Approximately 70% of antibody producing cells are is located in the digestive system.
Microbiota vs. Microbiome	The gut microbiota is a diverse ecosystem consisting of bacteria, archaea, viruses, protists and fungal communities (mycobiome) living in the human gut. Microbiome refers to the collection of genomes from all microorganisms in a particular environment
Transient vs. Resident Strain	Supplementary probiotics are transient strains. There is currently no evidence that supplementary probiotics can permanently colonize in the gut as resident strains resist colonization by transient strains. Transient probiotics strains may have numerous beneficial health effects by positively interacting with the immune system or stimulating growth of beneficial resident strains.
Alpha-Diversity	Represents the number of species and the proportion in which each species is represented in the microbiota. A high alpha diversity is present when there is a high number of species and their quantities are alike.
Beta-Diversity	Beta-diversity broadly reflects the species composition diversity between regional and local sites. The beta diversity measures the turnover of species between two regions in terms of gain or loss of species
Classes of probiotics	Definition
Lactic acid bacteria (LAB)	Nonpathogenic, nontoxigenic, Gram-positive, fermentative bacteria that are associated with the production of lactic acid from carbohydrates. LAB grow anaerobically, but unlike other anaerobes, most can grow in the presence of oxygen. Examples include *Lactobacillus* (ssp. *acidophilus, fermentum, plantarum, rhamnosus, casei, reuteri, gasseri*), *Streptococcus* (e.g. *salivarius*, *thermophilus*) and *Lactococcus*.
Bifidobacteria	*Bifidobacteria* are among the first microbes to colonize the human gastrointestinal tract. Examples include *Bifidobacterium bifidum*, *longum*, *animalis*, and *breve*. Bifidobacteria are not LAB. They are, however lactic acid producing bacteria (but through a very different metabolic pathway).
Spore-forming bacteria	Soil-based probiotics, also referred to endospores, are the dormant form of bacteria that are highly resistant to physical and chemical influences. Upon ingestion, these spores have a high survival rate through the stomach and germinate in the small intestine. Examples include *Bacillus* (e.g. *coagulans*, *subtilis*). Spore forming bacteria are not necessarily of soil origin. They can also be found in fermented foods.
Yeast	Examples include *Saccharomyces boulardii*.

**Fig. 1:**
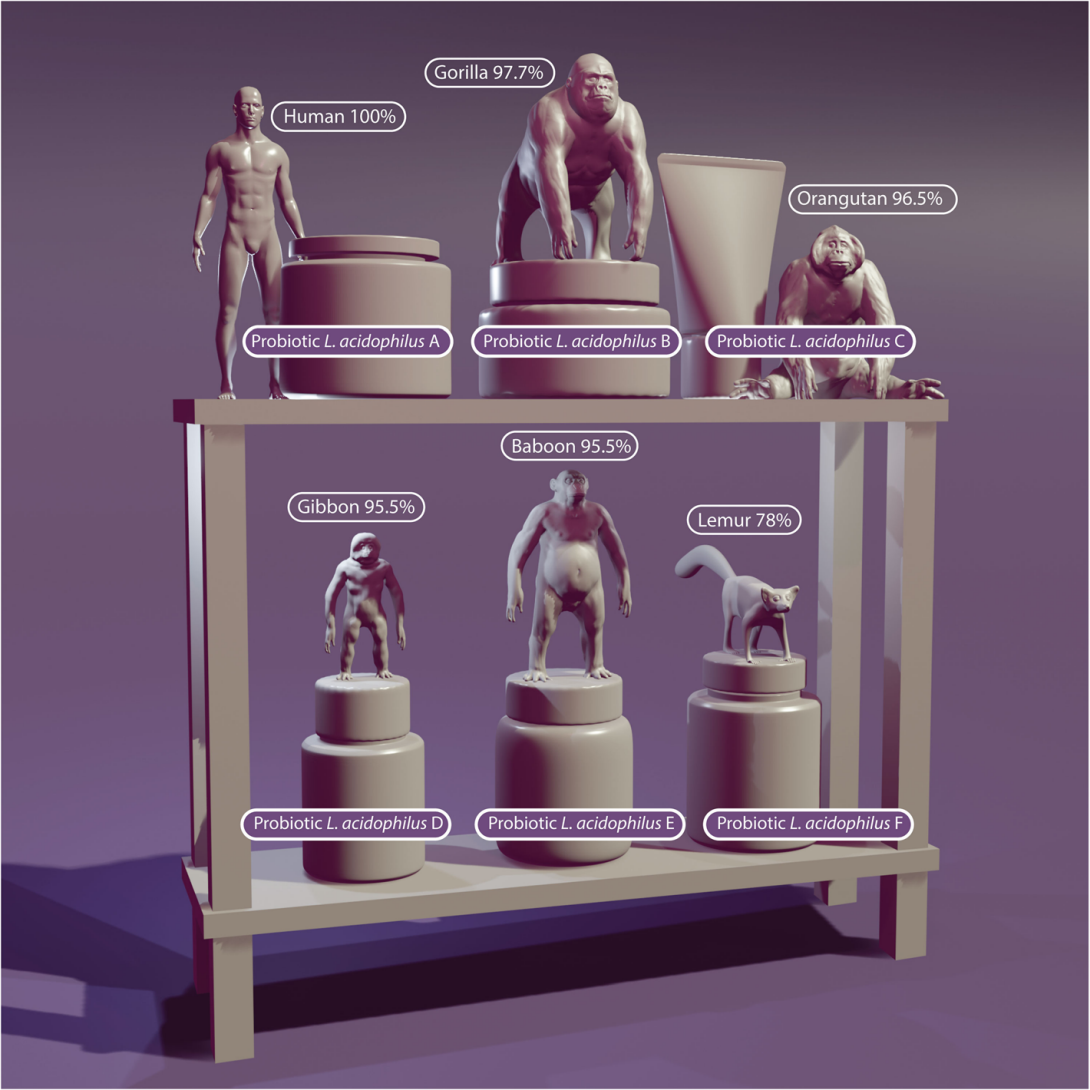
Probiotic benefits are strain specific and probiotics must be described as genus, species and strain, as genetic variation between the same genus and species can be as significant as the difference between a human and a lemur (illustration by Stephen Somers, Milwaukee, WI, USA)

The probiotic principle dates back to over 100 years ago. In 1908, Elie Metchnikoff [7] suggested that it would be possible to modify the microbiota in our bodies and replace harmful microbes with useful microbes. Reported health benefits of probiotics include modulation of the immune response, maintenance of the intestinal barrier, antagonism of pathogen adhesion to host tissue, and production of different metabolites such as vitamins, short-chain fatty acids (SCFAs), and molecules that act as neurotransmitters involved in gut–brain axis communication [8]. In the last several decades, research in the area of probiotics has progressed considerably and significant advances have been made in the selection and characterization of specific probiotic cultures. A growing number of dietary supplements containing probiotics are commercially available worldwide, and the number of products being marketed to improve the health and performance of athletes continues to increase substantially. To appropriately describe a probiotic, the genus, species, and strain of each live microorganism (see Table 2) must be detailed on a product label. Additionally, the product label should include the total estimated quantity of each probiotic strain at the end of the product’s shelf life, as measured by colony forming units (CFU) or live cells. Moreover, only a 70% DNA-DNA reassociation is needed for strains to be regarded as the same species [9]. The difference between a *Homo sapiens* and its most closely related species, the chimpanzee (*Pan troglodytes*) is 98.4%. Reassociation rates of humans with other primates like Gorilla (97.7%), Orangutan (96.5%), Siamang gibbon (95.5%), and the Hamadras baboon (92.7%) are also relatively high. Further, Lemur (78%) are still within the range for probiotics to be considered the same species (see Fig. 1). Analyzing potential health benefits of probiotics must occur on a strain level, and consumption of probiotic products only disclosing genus and species, but not the strain, on the label should be discouraged.

**Table 2:** Example illustrating the names of a bacterium (*L. rhamnosus* GG) at different taxonomic levels

Taxonomic level	Name
Domain	Bacteria
Phylum	Firmicutes
Class	Bacilli
Order	Lactobacillales
Family	Lactobacillaceae
Genus	*Lactobacillus*
Species	*Lactobacillus rhamnosus*
Strain	*Lactobacillus rhamnosus GG*

Probiotics are available commercially in capsule or tablet forms, as powder sachets, in the form of liquids and in specific foods such as yogurt and nutrition bars. While fermented foods, such as sauerkraut or kimchi, contain live microbes, they are currently not classified as probiotics, as those products have not been sufficiently studied for their health benefit as stipulated by the definition of probiotics. Stability concerns during manufacture and shelf-life limit food and supplement delivery forms. Probiotics exhibit strain-specific differences in their ability to colonize the gastrointestinal (GI) tract, clinical efficacy, and the type and magnitude of benefits to health in a range of different population cohorts [10]. The effects of probiotics in athletes have been less described in comparison to animal studies and human clinical conditions in the general population. However, the body of probiotic research in recreational and competitive athletes is expanding, including investigations in GI health, exercise performance, recovery, physical fatigue, immunity, and body composition.

### Role of diet and exercise on an athlete’s gut microbiome

Numerous factors such as age, genetics, drug use, stress, smoking, and especially diet can all affect the gut microbiome, influencing a complex ecosystem that is highly dynamic and individual [11–14]. In relation, physical activity has been an area of growing interest in gut microbiome research and appears to promote a health-associated microbiota. In the context of athletes, the present body of literature suggests their microbiota has several key differences in comparison to other populations, likely driven, in part, by exercise and diet. Indeed, several observational studies have investigated the difference in the composition of the gut microbiota between those who are highly physically active (including athletes) and a range of other populations. Reported results include that a higher abundance of health-promoting bacterial species [15–17], increased microbiome diversity [16, 18], and greater relative increases in metabolic pathways (e.g. amino acid and antibiotic biosynthesis and carbohydrate metabolism) and fecal metabolites (e.g. microbial produced SCFAs; acetate, propionate, and butyrate) are associated with enhanced fitness [17, 19].

The current evidence supports the role of exercise as an important behavioral factor that can affect qualitative and quantitative changes in the gut microbial composition with benefit to the host. Exercise appears to be able to enrich microbiota diversity [20–25], increase the *Bacteroidetes-Firmicutes* ratio [23], stimulate the proliferation of bacteria which can modulate mucosal immunity [26], improve barrier functions [27], and stimulate bacteria capable of producing substances that protect against GI disorders [28, 29]. Recent research provides further evidence for a role of exercise in shaping the microbiome, with elite runners having a greater abundance of *Veillonella* that appears to confer a metabolic advantage for endurance exercise by converting exercise-induced lactate to propionate. Pre-clinical studies with *Veillonella* show a 13% increase in endurance performance [30]. It is likely that the diverse, metabolically favorable intestinal microbiome evident in the elite athlete is the cumulative manifestation of many years of high nutrient intake and high degrees of physical activity and training throughout youth, adolescence and during adult participation in professional sports [31].

In researching the human gut microbiota, it is difficult to examine exercise and diet separately as this relationship is compounded by changes in dietary intakes that often are associated with physical activity (e.g., increased protein intake in resistance trained athletes or carbohydrate intake in endurance athletes and increased total energy and nutrient intake in general). Furthermore, comparing the microbiota of non-athletes to athletes and ascribing any observed differences to exercise alone is not advisable. Athletes generally consume a diet that differs from the general population that has implications for the composition of the gut microbiome.

Diet is an established modulator of gut microbiota composition, with significant change reported within 24 h of a dietary modification [32]. Various food components, dietary patterns, and nutrients all have the potential to alter considerably the growth of different gut microbial populations. Partitioning of individuals into enterotypes appears to be driven by whether their primary dietary patterns include high complex carbohydrate (*Prevotella*) or high fat/protein (*Bacteroides*) consumption [33]. Protein intake appears to be a strong modulator of the microbiota [20, 32, 34], with whey protein showing some potential benefits that need further study in humans [31, 35]. Carbohydrates are well known for their profound effect on the gut microbiota, with increased intake of dietary fiber associated with microbial richness and/or diversity [36, 37]. In athletes, higher intakes of carbohydrates and dietary fiber appear to be associated with increased abundance of *Prevotella *[17, 38]. The specific effects of fat on the gut microbiota is difficult to isolate, however, the types of fats consumed appear to be important [39]. Increased fat intake may promote higher concentrations of bile-tolerant bacteria (presumably because an extremely high fat intake is known to increase bile acid secretion) [32]. Further research is needed to determine the synthesis kinetics and clinical consequence of bile acids and their by-products during increased nutritional intake and metabolic demands during exercise.

**Fig. 2:**
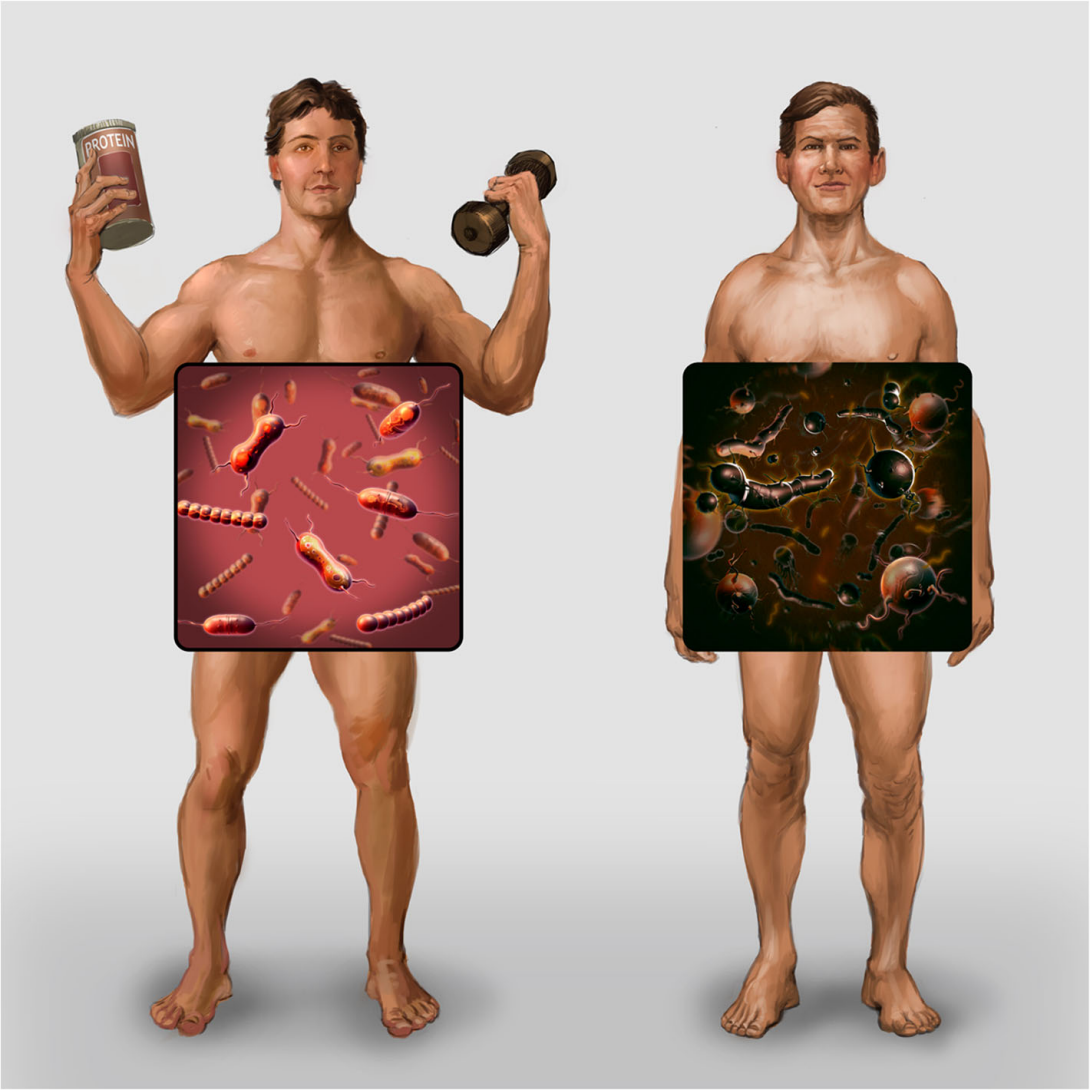
Early research indicates that gut bacteria reflect the activity level of its host**.** An athlete’s gut microbiota is different from a sedentary individual: increased diversity and greater abundance of health promoting bacterial species linked to exercise and increased protein intake (illustration by Stephen Somers, Milwaukee, WI, USA)

Based on the current body of evidence, the athlete gut microbiome may possess a functional capacity that is primed for tissue repair and a greater ability to harness energy from the diet with increased capacity for carbohydrate, cell structure, and nucleotide biosynthesis [19]. This assertion reflects the significant energy demands and tissue adaptation that occurs during intense exercise and elite sport. It appears that being physically active is another important factor in the relationship between the microbiota and host metabolism. Intervention-based studies to delineate this relationship will be important and may provide further insights into optimal therapies to influence the gut microbiota, and its relationship with health and disease as well as athletic performance. Fig. 2 illustrates that an athlete’s gut microbiota is different from a sedentary individual with increased diversity and greater abundance of health promoting bacterial species linked to exercise and increased protein intake.

**Table:** 

**Key Points 1 – Role of diet and exercise on an athlete’s gut microbiome.**	
• Active individuals appear to display a higher abundance of health-promoting bacterial species and increased microbiota diversity.	
• Body composition and physical activity are positively correlated with several bacterial populations.	
• Overall exercise can enrich the microbiota diversity, increase the *Bacteroidetes-Firmicutes* ratio, stimulate the proliferation of bacteria which can modulate mucosal immunity, and improve barrier functions.	
• Diet is an established modulator of gut microbiota composition and activity, with marked changes in microbiota composition evident within 24 h of a dietary modification.	
• Protein intake appears to be a strong modulator of microbiota diversity, with whey protein showing some potential benefits that need further study in humans.	
• Higher intakes of carbohydrate and dietary fiber in athletes appear to be associated with increased abundance of *Prevotella.*	
• The specific effects of fat on the gut microbiota is difficult to isolate, however, the types of fats consumed appear to be important.

### Benefits of probiotic supplementation in athletes

Strenuous and prolonged exercise places stress on the GI tract that increases the likelihood of multiple symptoms associated with a disturbed gut microbiota and decreased performance [40], including abdominal cramping, acid reflux (heartburn), nausea, vomiting, diarrhea, and permeability of the gut that may precipitate systemic endotoxemia [41]. As a major gateway for pathogen entry, the GI tract is heavily protected by the immune system. Modulation of the immune system to increase defenses against upper respiratory tract infection (URTI) is the potential benefit of probiotics for athletes that has been most extensively researched [40]. The microbiome may also have indirect functional influence on various indices of exercise performance and recovery [42–46]. Therefore, probiotics as functional modulators of the microbiome can potentially promote health, exercise adaptation, and performance in athletes.

Probiotics may regulate the mucosal immune response [47], improve the activity of macrophages [48] and modulate the expression of the genes associated with macrophage activity. Probiotics may also interact with Toll-like receptors (TLRs) and downregulate the expression of nuclear factor (NF)-κB and pro-inflammatory cytokines [49, 50]. Additionally, levels of anti-inflammatory cytokines and immunoglobulins, immune cell proliferation, and production of pro-inflammatory cytokines by T cells may be modulated following probiotic supplementation [51, 52]. However, it is often difficult to study athletes during training and competition, and a wide range of interactions between diet, physical activity and other lifestyle stresses needs to be considered. Understanding whether probiotics play a role in athletic performance is of particular interest to athletes who work to improve their results in competition as well as reduce recovery time during training. Moreover, this knowledge may be relevant and of direct benefit to general human health.

The study of probiotic supplementation in athletes and physically active individuals is quite new with the first study in humans published by Clancy et al. [53]. Over the last 13 years, the popularity and number of publications has increased substantially (see Table 3). The number of products containing probiotics directed towards those that exercise is increasing.

**Table 3:** Probiotic studies in an athletic population: performance, immune and GI health

Reference	Subject group	Sex and age (M ± SD)	Supplementation	Treatment duration	Exercise	Diet	Performance Benefit	Immune or GI Benefit
Clancy et al. (2006) [53]	Healthy recreational athletes (*n* = 18), Fatigued recreational athletes (*n* = 9)	11 M / 7 F 16–37 y 6 M / 3 F 17–40 y	*L. acidophilus* (LAFTI®L10), capsules, 2 × 10^10^ CFU Daily	4 weeks	Not reported	Not reported	Not assessed	T cell deficit was reversed (increased secretion of IFNƴ from T cells) following probiotic supplementation
Moreira et al. (2007) [54]	Non-elite Marathon runners (*n* = 141)	62 M / 8 F in treatment group 39 ± 9 y	*L. rhamnosus* GG (LGG), milk-based drink, 4 × 10^10^ CFU Daily	12 weeks	Running During pollen season & 2003 Helsinki City Marathon	Subjects instructed to refrain from eating food containing probiotics	Not assessed	No effects on symptoms of atopy or asthma
Kekkonen et al. (2007)* [55] *Same subjects as Moreira et al *(2007)* [54]	Non-elite Marathon runners (*n* = 141)	62 M / 8 F in treatment group 39 ± 9 y	*L. rhamnosus* GG (LGG), milk-based drink, 4 × 10^10^ CFU Daily	12 weeks	Running During pollen season & 2003 Helsinki City Marathon	Subjects instructed to refrain from eating food containing probiotics	Not assessed	No effect on respiratory infections or GI episodes. Shortened GI stress post marathon
Tiollier et al. (2007) [56]	French commando cadets (*n* = 47)	47 M 21 ± 0.4 y	*L. casei* DN- 1 14 00 1, milk-based drink during training (dose not indicated) Daily	3 weeks	Military training for 3 weeks followed by a 5-day combat course	Military ration. No fermented dairy products	Not assessed	No effect on respiratory tract infections
Cox et al. (2010) [57]	Elite male distance runners (*n* = 20)	20 M 27.3 ± 6.4 y	1.2 × 10^10^ CFU *L. fermentum* VRI-003 (PCC) Daily	16 weeks	Running (winter training)	Not reported	No changes in running performance	Significant reduction in respiratory episodes and severity
Martarelli et al. (2011) [58]	Amateur cyclists (*n* = 24)	24 M 32.03 ± 6.12 y	*L. rhamnosus* IMC 501®, *L. paracasei* IMC 502® 1 × 10^9^ CFU Daily	4 weeks	Intense physical activity	Diets proportionally equivalent in macro and micronutrient quantity, containing 100% of the RDA for all nutrients	Not assessed	Reduced exercise induced oxidative stress
Gleeson et al. (2011) [59, 60]	Recreationally active endurance athletes (*n* = 84)	54 M / 30 F 27.0 ± 11.6 y	*L. casei* Shirota (LcS), 6.5 × 10^9^ CFU 2x daily	16 weeks	Running (winter training, normal training load)	Consumption of supplements, additional probiotics, or any fermented dairy products were not permitted during the study period	Not assessed	Significant reduction in frequency of URTI
West et al. (2011) [61]	Competitive cyclists (*n* = 80)	64 M / 35 F 35 ± 9 and 36 ± 9 y	*L. fermentum* (PCC®) 1 × 10^9^ CFU Daily	11 weeks	Cycling (winter training, normal training load)	Subjects were asked to maintain a normal diet and refrain from eating probiotic or prebiotic enriched foods or supplements	No effect on peak power or VO2 max	Significant reduction in URTI (duration and severity) in males. No effect in females
Välimäki et al. (2012) [62]	Marathon runners Placebo (*n* = 58), Probiotic (*n* = 61)	105 M / 14 F 40 (23–69) y 40 (22–58) y	*L. rhamnosus* GG (LGG), 4 × 10^10^ CFU Daily	12 weeks	Running training; marathon run	Instructed to refrain from eating food containing probiotics and advised to follow normal dietary habits	Not assessed	No effects on serum LDL or antioxidant levels
Lamprecht et al. (2012) [63]	Endurance trained men (triathletes, runners, cyclists) (*n* = 23)	23 M 37.6 ± 4.7 y	Multispecies probiotic (*B. bifidum* W23, *B. lactis* W51, *E. faecium* W54, *L. acidophilus* W22, *L. brevis* W63, and *L. lactis* W58, 1 × 10^10^ CFU Daily	14 weeks	Normal training load	7-dayfood record. Instructed to maintain their habitual diet	No effect on VO2 max, maximum performance	Significant reduction in Zonulin (marker of gut permeability)
Gleeson et al. (2012) [64]	Highly active individuals (*n* = 66)	28 M / 38 W 23.9 ± 4.7 y	*L. salivarious*, 2 × 10^10^ CFU Daily	16 weeks	Endurance-based physical activities (spring training)	Consumption of supplements, additional probiotics, or any fermented dairy products was not permitted	Not assessed	No effect on frequency, severity and duration of upper respiratory tract infections
Grobbelaar et al. (2012) [65]	Moderately active individuals (*n* = 50)	50 M 18–30 y	*Bifidobacterium* and *Lactobacillus* strains (dose not indicated) Daily	6 weeks	Moderately active as defined by ACSM and CDC	Nutritional supplementation prohibited	Not assessed	No significant increases in performance related blood markers
West et al. (2012) [66]	Active individuals (*n* = 22)	22 M 33.9 ± 6.5 y	Multi-strain probiotic (4.6 × 10^8^ CFU *L. paracasei* subs p*aracasei* (*L. casei* 431®), 6 × 10^8^ CFU *B. animalis* ssp. *lactis* (BB-12®), 4.6 × 10^8^ CFU *L. acidophilus* LA-5, 4.6 × 10^8^ CFU *L. rhamnosus* GG Daily	3 weeks	Recreational cycling	Not reported	Not assessed	No effect on measures of systemic or mucosal immunity including gut permeability
Salarkia et al. (2013) [44]	Adolescent endurance swimmer (*n* = 46)	46 F 13.8 ± 1.8 y	Multi-strain probiotic yoghurt (*L. acidophilus* SPP, *L. delbrueckii bulgaricus*, *B*. *bifidum*, and *S. salivarus thermnophilus*) 4 × 10^10^ CFU Daily	8 weeks	Swimming	Advised to refrain from other probiotic products	Significant improvement in VO_2_ max. No effect on swim times	Significant reduction in respiratory and ear infections. No effect on GI episodes
Charlesson et al. (2013) Abstract of 2012 IJSNEM Confer.	Male athletes (*n* = 8) (travelling to high risk travelers’ diarrhea countries)	8 M Age not reported	*L. acidophilus*, *B. lactis*, *L. rhamnosus* (dose not indicated) Daily	8 weeks	Normal training	Not reported	Not assessed	No effect on travelers’ diarrhea (TD). 50% of all athletes reported TD symptoms
Sashihara et al. (2013) [67]	University-student athletes (*n* = 44)	44 M Grp-1: 19.8 ± 0.9 y Grp-2: 19.9 ± 0.9 y	Grp-1: *L. gasseri* OLL2809 1 × 10^9^ CFU. Grp-2: alpha-lactalbumin 900 mg +: *L. gasseri* OLL2809 1 × 10^9^ CFU 3x daily	4 weeks	Normal training load	Not reported	No improvement in 1 h of cycle ergometer exercise performance	Prevented reduced natural killer cell activity due to strenuous exercise and elevated mood from a depressed state (POMS)
West et al. (2014) [68]	Active individuals (*n* = 465)	241 M / 224 F 35 ± 12 y / 36 ± 12 y	*B. animalis* subsp. *lactis* BI-04 2 × 10^10^ CFU, or *L. acidophilus* NCFM and *B. animalis* subsp. *lactis* BI-07 5 × 10^9^ CFU Daily	150 days (21.42 weeks)	Normal activity load (approx. 6 h per week)	Refrain from consumption of non-study probiotic or prebiotic supplements or foods during the study.	Not assessed	BI-04 reduced upper respiratory tract infection frequency. BI-07 + LA NCFM showed no effect. Probiotic treatments delayed URTI ~ 0.8 months
Haywood et al. (2014) [69]	Highly-trained rugby union players (*n* = 30)	30 M 24.7 ± 3.6 y	*L. gasseri* 2.6 × 10^9^ CFU, *B. bifidum* 0.2 × 10^9^, and *B. longum* 0.2 × 10^9^ CFU Daily	4 weeks	Normal training load (during the winter months)	Asked to maintain a normal diet and refrain from consuming probiotic and prebiotic enriched foods or supplements	Not assessed	Significant reduction in episodes of illness. No effect on illness severity
Shing et al. (2014) [46]	Runners (*n* = 10)	10 M 27 ± 2 y	Multispecies probiotic (*L. acidophilus*, *L. rhamnosus*, *L. casei*, *L. plantarum*, *L. fermentum*, *B. lactis*, *B. breve*, *B. bifidum*, and *S. thermophilus*) 4.5 × 10^10^ CFU Daily	4 weeks	Normal training load	Provided with a high glycemic index, low sucrose diet for the 26 h prior to each time to-fatigue run.	Significant increase in run time to fatigue in the heat	No effects on inflammation or GI markers
Aghaee et al. (2014) [70] *Abstract*	Athletes (*n* = 16)	16 M 19–25 y	Probiotic (type and dose not indicated) Daily	30 days	Normal training load	Not reported	Not assessed	Probiotic treatment significantly increased monocyte levels in comparison to placebo control
Georges et al. (2014) PILOT [71]	Resistance-trained individuals (*n* = 10)	10 M 22.0 ± 2.4 y	*B. coagulans* GBI-30, 6086 (BC30), 5 × 10^8^ CFU plus 20 g of casein 2x daily	8 weeks	Periodized resistance training (4x per week)	Macronutrients were controlled to 50% carbohydrate, 25% protein, and 25% fat between groups.	Trend to increase vertical jump power (not significant).	Not assessed
Narimani-Rad et al. (2014) [72]	Professional bodybuilding athletes (*n* = 14)	14 M 20–55 y	Multi-strain probiotic (*L. casei* 5.1 × 10^9^ CFU/g, *L. acidophilus* 2 × 10^9^ CFU/g, *L.* C. 5.1 × 10^9^ CFU/g, *L. bulgaricus* 2 × 10^8^ CFU/g, *B. breve* 2 × 10^10^ CFU/g, *B. longum* 7 × 10^7^ CFU/g, *S. thermophilus* 5.1 × 10^9^ CFU/g) Daily	30 days	Normal training load	Not reported	Not assessed	Stimulated thyroid activity. Significant increase in T_4_ and significant decrease TSH levels. No significant difference in T_3_ levels
Muhamad & Gleeson (2014) [73]	Active University students (*n* = 11)	11 (sex not reported) 22 ± 1 y	14 strain probiotic (*L. acidophilus*, *L. delbrueckii* ssp. *bulgaricus*, *L. lactis* ssp. *lactis*, *L. casei*, *L. helveticus*, *L. plantarum*, *L. rhamnosus*, *L. salivarius* ssp. *salivarius*, *B. breve*, *B. bifidum*, *B. infantis*, *B. longum*, *B. subtilis*, and *S.* *thermophilus*.) 6 × 10^9^ CFU Daily	30 days	Not reported	Not reported	No significant change in rating of perceived exertion and HR	No significant change in salivary antimicrobial proteins (a measure of mucosal protection)
Salehzadeh (2015) [45]	Endurance athletes (*n* = 30)	30 M 21 y	200 ml of probiotic yogurt drink *S. thermophilu*s or *L. delbrueckii* ssp. *bulgaricus* 1 × 10^5^ CFU/g Daily	30 days	Intense aerobic training	Not reported	Significant increase in VO2 MAX and aerobic power	Significant decrease in serum CRP, significant increase in HDL
O’Brien et al. (2015) [74]	Male and female runners (*n* = 67)	Not reported 18–24 y	Kefir beverage (probiotic strain and amount not indicated) 2x week	15 weeks	Marathon training program	Not reported	No effect on 1.5 mile run test times	Attenuated increase in inflammation (serum CRP)
Gill et al. (2016a) [75]	Endurance-trained runners (*n* = 8)	8 M 26 ± 6 y	*L. casei* 10 × 10^10^ CFU Daily	7 days	Running exercise in hot ambient temperature	Refrained from alcohol and caffeine for 72 h and exercise for 24 h before preliminary testing sessions and each experimental trial	No difference in exercise performance on a treadmill test and perception of effort	No improvement in salivary antimicrobial protein (mucosal immune protection) or cortisol status over placebo
Gill et al. (2016b) [76]	Endurance-trained runners (*n* = 8)	8 M 26 ± 6 y	*L. casei* 10 × 10^10^ CFU Daily	7 days	Running exercise in hot ambient temperature	Consumption of other probiotics was prohibited outside the study protocol	Not reported	Did not prevent increases in external heat stress-induced circulatory endotoxin concentration or plasma cytokine profile compared with placebo
Jäger et al. (2016) [42]	Recreationally-trained individuals (*n* = 29)	29 M 21.5 ± 2.8 y	*B. coagulans* GBI-30, 6086 (BC30), 1 × 10^9^ CFU plus 20 g of casein protein Daily	2 weeks	Muscle-damaging single leg training bout	Subjects provided a standardized meal prior to exercise bout. Three-day dietary recalls were collected	Significantly increased recovery and decreased soreness. Non-significant trend to increase power	Not assessed
Jäger et al. (2016) [43]	Resistance-trained men (*n* = 15)	15 M 25 ± 4 y	*B. breve* BR03 5 × 10^9^ live cells (AFU) & *S. thermophilus* FP4 5 × 10^9^ live cells (AFU) Daily	3 weeks	Normal training up until 72 h preceding muscle-damaging elbow flexor exercise challenge	Refrain from any nutritional supplements or ergogenic aids	Improved isometric average peak torque production and range-of-motion during acute recovery	Significant decrease in marker of inflammation (IL-6)
Roberts et al. (2016) [77]	Recreational triathletes (*n* = 30)	25 M / 5 F 35 ± 1 y	Multi-strain pro/prebiotic/antioxidant 30 × 10^9^ CFU per day containing 10 × 10^9^ CFU *L. acidophilus* CUL-60 (NCIMB 30157), 10 × 10^9^ CFU *L. acidophillus* CUL-21 (NCIMB 30156), 9.5 × 10^9^ CFU *B. bifidum* CUL-20 (NCIMB 30172) and 0.5 × 10^9^ CFU *B. animalis* subsp. *lactis* CUL-34 (NCIMB 30153)/55.8 mg fructooligosaccharides/ 400 mg alpha-lipoic acid, 600 mg N-acetyl-carnitine Daily	12 weeks	Progressive triathlon training program	Maintained habitual dietary intake. Required not to consume any other nutritional supplement	No significant difference in race times	Significant reduction in endotoxin levels
Strasser et al. (2016) [78]	Trained athletes (*n* = 29)	13 M / 16 F 26.7 ± 3.5 y	Multi-species probiotic (*B. bifidum* W23, *B. lactis* W51, E. *faecium* W54, *L. acidophilus* W22, *L. brevis* W63, and *L. lactis* W58) 1 × 10^10^ CFU/g Daily	12 weeks	Winter training	Maintain normal diet and avoid anti-inflammatory drugs, antibiotics, additional probiotics and dietary supplements	Did not benefit athletic performance	Limited exercise-induced drops in tryptophan levels and reduced the incidence of URTI
Michalickova et al. (2016) [79]	Elite athletes (badminton, triathlon, cycling, alpinism, karate, savate, kayak, judo, tennis and swimming) (*n* = 39)	29 M / 10 F 23.15 ± 2.6 y	*L. helveticus Lafti* L10, 2 × 10^10^ CFU Daily	14 weeks	Normal training load (during winter)	Subjects maintained normal diet and were asked to avoid fermented milk products and immunomodulatory supplements	No significant differences in exercise performance	Significant reduction in duration of URTI episodes and decreased symptoms in elite athletes
Gleeson et al. (2016) [80]	College athletes (*n* = 243)	142M / 101F 20.4 ± 0.2 y	Fermented milk beverage containing *L. casei* Shirota, 6.5 × 10^9^ CFU 2x daily	20 weeks	Normal training load	Supplements that might influence immune function and additional probiotics or fermented dairy were not permitted	Not assessed	Significant reduction in cytomegalovirus and Epstein Barr virus antibody titres, benefiting immune status
Michalickova et al. (2017)	Elite athletes (badminton, triathlon, bicycling, athletics, karate, kayaking, and judo) (*n* = 30)	24 M / 6 F 23.6 ± 1.9 y	*L. helveticus* Lafti L10, 2 × 10^10^ CFU Daily	14 weeks	Normal training load (winter training)	Subjects maintained normal diet and were asked to avoid fermented milk products and immunomodulatory supplements	Not assessed	Supported humoral and mucosal immunity by preserving total salivary Immunoglobulin A level
Gepner et al. (2017)	Soldiers from elite combat unit (*n* = 26)	26 M 20.5 ± 0.8 y	*B. coagulans* GBI-30 (BC30) 1.0 × 10^9^ CFU and HMB 3 g Daily	40 days	Strenuous military training 40 days	No additional dietary supplements nor consumtion any androgens or other performance-enhancing drugs	Not assessed	Combined supplementation attenuated IL-6 and IL-10 response and maintained muscle integrity
Marshall et al. (2017) [81]	Marathon competitors (*n* = 32)	26 M / 6 F 23–53 y	PRO-grp: Multi-strain capsule; *L. acidophilus* CUL-60 10 × 10^9^ CFU, and *L. acidophillus* CUL-21 (NCIMB 30156) 10 × 10^9^ CFU), *B. bifidum* CUL-20 9.5 × 10^9^ CFU and *B. animalis* subsp. *lactis* CUL-34 0.5 × 10^9^ CFU, and 55.8 mg fructooligosaccharides. PGLn-grp: *L. acidophilus* CUL-60 (NCIMB 30157) 2 × 10^9^ CFU, *L. acidophilus* CUL-21 (NCIMB 30156) 2 × 10^9^, *B. bifidum* CUL-20 (NCIMB 30172) 0.5 × 10^9^ CFU, *B. animalis* subsp. *lactis* CUL-34 (NCIMB 30153) 0.95 × 10^9^ CFU, *L.* salivarius CUL61 (NCIMB 30211) 5 × 10^9^ CFU, and each 5-g dose also contained 0.9 g L-glutamine. Daily	12 weeks	Marathon training; Marathon race	Not permitted to consume any other commercial supplementation that conflicted with the study parameters	No difference in marathon time to completion compared to control group	No change in immuno-stimulatory heat shock protein (eHsp72) concentrations
Toohey et al. (2018) [20]	Soccer and volleyball Division I college athletes (*n* = 23)	23 F 19.6 ± 1.0 y	*B. subtilis* (DE111) 5 × 10^9^ CFU Daily	10 weeks	Offseason resistance training program	No dietary restrictions were placed on the athletes besides abstaining from other supplement use	No effect on physical performance parameters	Significant reduction in body fat percentage
Brennan et al. (2018) [82] Abstract of 2018 ACSM Confer.	Endurance athletes (*n* = 7)	(sex not reported) 31 ± 6.1 y	*L. salivarius* (UCC118) (dose not indicated) Daily	4 weeks	Not reported	Not reported	Not assessed	Exercise-induced intestinal hyperpermeability was attenuated
Townsend et al. (2018) [83]	Division I Baseball Players (*n* = 25)	25 M 20.1 ± 1.5 y	*B. subtilis* (DE111) 1 × 10^9^ CFU Daily	12 weeks	Offseason training	Three-day food logs collected on weeks 1, 9 and 12.	No effect on physical performance or body composition	TNF-α concentrations were significantly lower compared to placebo
Antonio et al. (2018) [84]	Active men and women (*n* = 20)	6 M/ 14 F 30 ± 8 y	*B. breve* BR03 5 × 10^9^ CFU and *S. thermophilus* FP4 5 × 10^9^ CFU Daily	6 weeks	Normal training load (aerobic and/or resistance training)	Subjects were instructed to not alter their diet	No effect on body composition	Not assessed
Huang et al. (2018) [85]	Healthy adults without professional athletic training (*n* = 16)	16 M 20–40 y	*L. plantarum* TWK10 1 × 10^11^ CFU Daily	6 weeks	Not reported	Normal diet maintained and no consumption of any other nutritional supplements	Improved endurance performance and blood glucose concentration in a maximal treadmill running test	Not assessed
Carbuhn et al. (2018) [86]	Division I collegiate female swimmers (*n* = 17)	17 F Age not reported	*B. longum 35,624,* 1 × 10^9^ CFU Daily	6 weeks	Offseason training	Three-day food logs collected at baseline and weeks 3 and 6.	No effect on aerobic/anaerobic swim time trials and force plate vertical jump	No effect on cytokine and gastrointestinal inflammatory markers and salivary IgA levels
Huang et al. (2019) [87]	Healthy adult triathletes (*n* = 34)	Study 1: 18 M, 20.2 ± 0.7 y Study 2: 16 M, 22.3 ± 1.2 y	*L. plantarum* PS128 3 × 10^10^ CFU Daily	Study 1: 4 weeks Study 2: 3 weeks	Sprint triathlon (swimming 750 m, biking 20 km, running 5 km).	Before race: 595 kcal (24 g PRO, 16 g FAT, 90 g CHO). In race: 30–40 g CHO and 500–1000 ml water per hour.	Attenuated post-triathlon performance declines. No effect on body composition.	Reduced post-race inflammatory cytokines, reduced oxidative stress, increased plasma BCAA levels.
Pugh et al. (2019) [88]	Health adult marathon runners (ran marathon race quicker than 5 h within the previous 2 years; *n* = 24)	20 M / 4 F 34.8 ± 6.9 y	*L. acidophilus (CUL60 and CUL21), B. bifidum (CUL20), B. animalis subs p. Lactis (CUL34)* *> 25 billion CFU daily in total, no information on individual strains*	4 weeks (pre-race)	Marathon race	Before race: standardized high CHO, low fiber diet. In race: 60 mL CHO gel with 200 mL (15 min before start, 40 min post and every 20 min for the remainder of the race.	No difference in race times.	GI symptom severity during the final third was significantly lower.
Pumpa et al. (2019) [89]	Elite rugby union athletes (*n* = 19)	19 M 27.0 ± 3.2 y	*L. rhamnosus, L. casei, L. acidophilus, L. plantarum, L. fermentum, B. lactis, B. bifidum, S. thermophilus* 120 billion CFU daily in total, no information on individual strains 500 mg *S. boulardi* (added during stage 3)	17 weeks	27-weeks, divided into three stages: 1) control period (10 weeks); 2) domestic competition (7 weeks); 3) international competition (10 weeks).	A national training camp and 3 domestic games (stage one), 6-weeks of domestic competition (stage two), and 8-weeks of international competition (stage three).	Not assessed	No effect on salivary Immunoglobulin A. Salivary cortisol increased. Increase in salivary alpha-amylase levels during stage 3.
Vaisberg et al. (2019) [90]	Amateur marathon runners with previous history of post-race URTI (*n* = 42)	42 M 39.5 ± 9.4 y	Fermented milk beverage containing *L. casei* Shirota, 4 × 10^10^ CFU Daily	30 days (pre-race)	Marathon race	Unknown	Not assessed	Improved airway and systemic immune and inflammatory responses post-marathon. No significant effect on URTI.

#### The effect of probiotic supplementation on performance

Research specifically designed to investigate the effect of probiotic supplementation on performance has been less common and overall the results are mixed. Earlier studies that reported performance outcomes generally had primary aims related to immunity and GI health. Of the 24 studies that assessed some metric of athletic performance, 17 reported a null effect, while 7 reported significant improvement. However, more recent research indicates that probiotic supplementation can promote improvements in exercise performance through various pathways in athletes and physically active individuals using discrete strains of probiotics.

Some studies have used single probiotic strain interventions. For example, in a 16-week study investigating the effect of *Lactobacillus fermentum* VRI-003 on the immunity in 20 elite male distance runners, measures of performance (which included training duration, intensity, and VO_2_ max) did not change significantly [57]. Similarly, in 80 competitive cyclists, 11 weeks of supplementation with *L. fermentum (PCC®)* had no effect on peak power or VO_2_ max [61]. Four weeks of supplementation with *Lactobacillus gasseri* OLL2809 and alpha-lactalbumin in 44 university-student athletes did not improve cycle ergometer performance [67]. Gill et al. [75] did not find a difference in perception of effort during a treadmill test in eight male endurance-trained runners who supplemented with a high-dose of *Lactobacillus casei* (10 × 10^10^ CFU). Finally, in 39 elite athletes from various sports, 14 weeks of *Lactobacillus helveticus Lafti* L10 supplementation during the winter did not elicit significant differences in exercise performance as measured by VO_2_ max, treadmill performance time, maximal heart rate and heart rate recovery [79]. The single strain interventions used in these five studies did not produce an aerobic performance benefit.

Null findings were similarly reported in several studies investigating the effects of multi-strain probiotics on aerobic performance. For instance, in endurance-trained men, 14 weeks of a multi-species probiotic had no effect on VO_2_ max and maximum performance [63]. In a study designed to determine the effects of a 30-day period of supplementation with a 14-strain probiotic at rest, and in response to an acute bout of prolonged cycling exercise for 2 h at 60% VO_2_max in 11 active, healthy adults there was no significant change in rating of perceived exertion and heart rate [73]. In another study assessing the effects of a multi-strain probiotic (along with 55.8 mg fructooligosaccharides, 400 mg alpha-lipoic acid, 600 mg N-acetyl-carnitine) in 30 recreational athletes over 12 weeks of progressive triathlon training no significant differences were found in race times [77]. Marshall et al. [81] investigated the effects of a multi-strain probiotic for 12 weeks of marathon training in a group of 32 marathon competitors and found no difference in marathon time to completion compared to the control group.

However positive results were reported in thirty endurance athletes supplementing with a yogurt drink, either containing *Streptococcus thermophilus* or *Lactobacillus delbrueckii ssp*. *bulgaricus* or no probiotics over 30 days during intense aerobic training. There was a significant increase in VO_2_max and aerobic power in the Cooper aerobic test [45]. In thirty-three trained athletes, 12 weeks of winter training supplementation with a multi-species probiotic did not benefit athletic performance; however, the training load (hours per week) was higher in those who supplemented with the probiotic blend vs. the placebo group [78]. One explanation for these findings could be that probiotics may enable better performance capabilities and training adherence when the risk of URTI development is reduced, as individuals with fewer episodes of infections such as common colds are able to train more often and harder. Further, Strasser et al. [78], noted that the multi-species probiotic limited exercise-induced reductions in circulating tryptophan concentration. Higher serum tryptophan levels may enhance the tryptophan transport into the brain and support serotonin metabolism, which can influence an individual’s sensation of fatigue and thus potentially affect training adherence and performance [91]. Interestingly, VO_2_max was positively correlated with pre-exercise serum tryptophan levels at a moderate magnitude, supporting a role of tryptophan metabolism in training performance.

Huang et al. [85], found increased endurance performance and elevated blood glucose concentration following exercise-to-exhaustion after 6 weeks of high dose (1 × 10^11^ CFU) *Lactobacillus plantarum* TWK10 (a plant *Lactobacillus* strain isolated from Taiwanese pickled vegetables) supplementation in healthy male adults. However, as these were untrained males and no aerobic exercise intervention was reported in this study, these data should be interpreted conservatively in relation to endurance athletes. These results might be explained by an anti-inflammatory effect from *L. plantarum* TWK10 [92] on skeletal muscle and improvement in energy harvest, possibly related to glycogenesis regulation for exercise demand. Interestingly, *L. plantarum* KX041 can maintain intestinal permeability and exert antioxidant capacity [93]. Moreover, certain strains of *L. plantarum* activate cell growth signaling pathways in gut enterocytes which in turn increases protein metabolism in the gut [94]. Further, *L. plantarum* can rescue the shunted growth phenotype in malnourished mice by activating muscle, bone, and organ growth [95].

In a study investigating the effect of a multi-strain probiotic yogurt on performance in adolescent female endurance swimmers over 8 weeks, there was a significant improvement in VO_2_ max [44]. The improvement in VO_2_ max was attributed to the reduction in number and duration of URTI for athletes following intake of the multi-strain probiotic yogurt. In another study researching the effect of multi-strain probiotics Shing et al. [46] found 4 weeks of supplementation improved time to fatigue while running in the heat for ten male runners. While the mechanism for improvement was unclear, it was speculated that probiotics may exert small to large effects on GI structural integrity, endotoxin translocation and immune modulation that combine to enhance exercise performance. In contrast, a Kefir beverage (a naturally fermented milk beverage containing a defined mixed microbial culture of lactic acid bacteria and yeasts) consumed over 15 weeks of marathon training by sixty-seven male and female runners had no effect on 1.5 mile run test performance [74]. Currently, there are more studies showing a benefit for multi-strain probiotics in relation to performance measures compared to single-strain probiotics. While there are some encouraging results, a large majority of studies have found no effect on aerobic performance. It appears that some of the positive benefits of probiotic supplementation may be indirect by allowing for improved gut integrity or immune modulation. However, additional research is warranted including investigating potential performance outcomes beyond aerobic-based endurance exercise.

Other studies have explored the effect of probiotic supplementation in relation to resistance training on muscle recovery and body composition. A pilot study in ten subjects using resistance trained males supplemented 20 g of casein protein with or without *Bacillus coagulans* GBI-30, 6086 (BC30) for 8 weeks following a periodized resistance training program showed a trend to increase vertical jump power [71]. Jäger et al. [43] speculated that the potential improvement in vertical jump performance may have been related to improved muscle recovery through gut microbial modulation. In a follow up study, 20 g of casein protein co-administered with *B. coagulans* GBI-30, 6086 (BC30) or a placebo in recreationally-trained individuals for 2 weeks increased recovery and decreased soreness after a muscle-damaging single-leg training bout [43]. Furthermore, exercise-induced muscle damage was decreased as measured by serum creatine kinase, which may also indicate improved cellular integrity rather than damage per se. While not fully understood, candidate mechanisms of action included the production of digestive enzymes that are active under gut conditions (e.g. alkaline proteases) and these proteases can digest proteins more efficiently than the endogenous human proteases alone [43, 96, 97]. Further, *B. coagulans* GBI-30, 6086 enhances the health of the cells of the gut lining through improved nutrient absorption including minerals, peptides, and amino acids by decreasing inflammation and encouraging optimum development of the absorptive area of the villi [98]. In vitro, *B. coagulans* GBI-30, 6086 can increase protein absorption [99]. The combination of *B. coagulans* GBI-30, 6086 with casein protein may have acted synergistically to augment digestion and modulate absorption.

In fifteen resistance-trained men, 3 weeks of *Bifidobacterium breve* BR03 and *S. thermophilus* FP4 supplementation improved isometric mean peak torque production and range-of-motion during acute recovery after a muscle-damaging elbow flexor exercise challenge in comparison to a control group [42]. While mechanisms behind these observations were not described, these strains can have anti-inflammatory effects [100–102] and colonize in different areas of the GI tract. However, using the same strains and dose, Antonio et al. [84], failed to see a significant effect on body composition in highly-trained men and women over a longer, six-week period. In both of the above studies participants were not provided supplemental protein. Toohey et al. [103] investigated the effects of *Bacillus subtilis* DE111 probiotic supplementation on muscle thickness and strength, body composition, and athletic performance in Division I female volleyball and soccer athletes for 10 weeks of an offseason resistance training program. Both groups consumed a protein and carbohydrate recovery drink (consisting of 45 g carbohydrates, 20 g protein, and 2 g fat) immediately after each training session. Probiotic supplementation with the post-workout recovery drink yielded greater reductions in body fat and increases in fat free mass after 10 weeks of resistance training than a placebo. Although no performance advantages were observed, Toohey et al. [103], speculated that supplementation may have promoted improved dietary protein absorption and utilization, contributing to improvements in body composition by increasing dietary protein-induced thermogenesis and altering satiety signaling. It seems that several strains of lactic acid bacteria, including *L. gasseri* SBT 2055, *Lactobacillus rhamnosus* ATCC 53103, and the combination of *L. rhamnosus* ATCC 53102 and *Bifidobacterium lactis* Bb12, are effective at reducing fat mass in obese humans [104]. Additionally, other strains of *B. breve* have shown anti-obesity effects in both humans [105] and mice [106].

Townsend et al. [83], evaluated the effect daily *B. subtilis* (DE111) supplementation on physical and performance adaptations in Division I collegiate baseball players following 12 weeks of offseason resistance training. On training days, placebo or probiotic capsules were consumed immediately post-workout with a protein and carbohydrate recovery drink (consisting of 36 g carbohydrates, 27 g protein, and 2 g fat). There were no group differences observed between those who took the probiotic and placebo for any measure of strength, performance, or body composition. However, those athletes who did supplement with probiotics had significantly lower serum TNF-α concentrations than the placebo group. Elevations in TNF-α have been linked to suppressed protein synthesis, disordered sleep, and impaired muscular performance [107–109]. The null performance findings reported by Townsend et al. [83] and Antonio et al. [84] may have been the result of an inability for the probiotic supplement to modify healthy participants’ microbiomes. Indeed, the subjects in these two studies were young, healthy and highly active. In this regard, systematic reviews [110, 111] and an original investigation involving supplementation [112] of probiotic supplementation in adults indicate that probiotic supplementation is more likely to alter the microbiome composition of dysregulated microbiomes compared to healthy ones. While probiotic consumption may not alter microbiome composition, it can alter functionality by up regulation of gene expression and metabolic pathways. As noted for aerobic performance, it is also plausible that probiotic supplementation confers an indirect effect on performance and that the training, diet, and recovery of the individuals in some of these studies were optimal enough to mask any small additional benefits.

**Table:** 

**Key Points 2 – Probiotic Supplementation and Performance**	
• To date single-strain probiotic supplementation has produced a significant aerobic performance benefit in only one study.	
• Supplementation with multi-strain probiotics has been reported to increase VO2 max, aerobic power, training load, and time to exhaustion in several studies, but more studies have not found such an effect.	
• In response to muscle-damaging resistance exercise, probiotic supplementation (paired with protein) can expedite recovery and decrease soreness and other indices of skeletal muscle damage.	
• The effect of probiotic supplementation on body composition has been mixed and requires further research.	
• Probiotics supplementation as an ergogenic aid for performance enhancement requires further investigation and may be indirect via modulation of other systems.	

#### The effect of probiotic supplementation on the immune system

The mucosal lining of the GI tract represents the first-line-of-defense against invading pathogens and is an important interface with the host immune system. Exhaustive physical exercise negatively impacts immunity, reducing of the count and function of immune cells, such as natural killer (NK) cells and T lymphocytes. Pro-inflammatory cytokines such as IL-1, TNF-α and IFN-γ generally remain unchanged after prolonged exercise whereas the inflammation-responsive cytokine IL-6 and anti-inflammatory cytokines such as IL-10, IL-1ra, sTNFR increase markedly. The increase in IL-6 is not solely in response to inflammation in this situation as it also originates from contracting muscle and is associated with glycogen regulation. Gene expression in white blood cells is upregulated for most anti-inflammatory markers and downregulated for pro-inflammatory markers and TLR signaling. The anti-inflammatory hormone cortisol is also elevated [53, 57, 59, 113, 114]. Changes in immune health are associated with increased incidence of URTIs and disorders of the GI tract [46, 53] which have the potential to impair physical performance and/or cause an athlete to miss training or competition [115]. These conditions usually occur during competitive periods that are commonly represented by higher intensities and greater volumes of exercise [116], affecting the athlete’s health and impairing physical performance when needed most [115]. In this context, interventions that prevent or mitigate these conditions can indirectly improve physical and competition performance. Among the nutritional supplements used in modulation of the immune response of athletes, probiotics are noteworthy [92].

Probiotics appear to augment intestinal communication between the host immune system and commensal bacteria to establish mutualistic benefits. The roles of microbial-derived SCFAs, particularly butyric acid in the colon, are important in mucosal homeostasis through regulation of epithelial turnover and induction of regulatory T (Treg) cells [117]. Beyond the GI tract, probiotics have an immunomodulatory effect through the common mucosal immune system, in which cells from inductive sites (e.g., Peyer’s Patches in the intestines) translocate to mucosal surfaces following interaction with antigen-presenting cells [118].

Research investigating the effects of probiotics on immune outcomes have been the most prevalent type of research in athletic populations. Of the 22 studies reviewed in this Position Stand that assessed the effect of probiotics on outcomes related to the immune system, 14 reported significant improvement, whereas 8 reported no effects.

Of particular relevance to athletes is the reduction in incidence and/or severity of symptoms from illnesses like URTI. In a large study of 465 active individuals who had a normal activity load of approximately 6 h per week, West et al. [68] compared a single strain treatment consisting of *Bifidobacterium animalis* ssp. *lactis* Bl-04 and double-strain probiotic consisting of *Lactobacillus acidophilus* NCFM and *B. animalis* subsp. lactis Bi-07 to placebo over a 150-day intervention. Daily *B. animalis* ssp. *lactis* Bl-04 supplementation for 150 days was associated with a 27% reduction in the risk of any URTI episode compared to placebo supplementation. Supplementation with the double-strain probiotic resulted in a 19% decrease of URTI risk, although this was not statistically significant. Moreover, both probiotic supplement groups exhibited a ~ 0.8-month delay in time to illness. Importantly, healthy active individuals with a lighter training load, and presumably at a lower risk for URTIs, also appeared to benefit from a probiotic supplement.

The majority of studies that have investigated the potential benefits of probiotics on URTIs have been conducted in endurance athletes with generally high training loads. For example, Cox et al. [57] studied the effect of *L. fermentum* VRI-003 (PCC) over 16 weeks of winter training in 20 elite male distance runners on incidence of illness and infection. Probiotic supplementation significantly reduced URTI incidence and severity compared to placebo. Specifically, those in the treatment group reported less than half the number of days of respiratory illness symptoms compared to the control group during the intervention. While not significant, there was a trend for enhanced T-lymphocyte function, which may be in part responsible for the immunological benefits. Similarly, Gleeson et al. [60] examined the effects of *Lactobacillus casei* Shirota during 4 months of winter training in endurance-based recreational athletes and observed a significant reduction in URTIs compared to placebo. In addition, salivary IgA concentration was significantly higher in those consuming the probiotic. However, severity and duration of symptoms were similar between the treatment and placebo groups. Supplementation with the same strain 30 days prior to a marathon race resulted in improved systemic and airways immune responses, and showed a trend toward improved incidents and duration of URTI post-marathon [90]. In competitive cyclists, West et al. [61] reported reduced severity of self-reported symptoms of lower respiratory illness and use of cold and flu medication over an 11-week winter training period with *L. fermentum* (PCC®) compared to placebo. Interestingly, this effect was only noted in males and not females. Strasser et al. [78] examined the effect of 12 weeks of treatment with a multi-strain probiotic on the incidence of URTIs and metabolism of aromatic amino acids after exhaustive aerobic exercise in highly trained athletes during the winter. Daily supplementation with probiotics reduced the incidence of URTI compared to placebo. In addition, supplementation limited exercise-induced reductions in tryptophan levels, which may reduce the risk of developing an infection.

Beyond studies investigating traditional endurance athletes with high aerobic training loads, probiotic supplementation has also been examined in other athletes with varying demands. For instance, Salarkia et al. [44] reported that 8 weeks of supplementation with a multi-strain probiotic yogurt reduced the number of episodes of URTIs in adolescent female swimmers compared to the same yogurt without probiotics. Haywood et al. [69] investigated the effect of a multi-strain probiotic over 4 weeks in 30 elite union rugby players to determine effectiveness on the number, duration and severity of infections. The probiotic group had lower incidence of infection-related symptoms compared to placebo, although there was no difference in the severity of the symptoms between the two treatment groups. In a study of an eclectic group of elite athletes training in badminton, triathlon, cycling, alpinism, athletics, karate, savate, kayak, judo, tennis, and swimming, Michalickova et al. [79] studied the effects of *L. helveticus* Lafti L10 over 14 weeks during the winter. Athletes all had high training loads of > 11 h per week and were winners of the national or European and world championships in their categories and sport. Supplementation with the probiotic significantly reduced the length of URTI episodes and lowered the number of symptoms per episode compared to placebo. Moreover, there was a significant increase of CD4+/CD8+ (T helper/T suppressor) cells ratio in the probiotic group. Previously, this ratio has been noted as an index sensitive to high training loads and was decreased after strenuous physical activity [36, 119]. In addition, low CD4+/CD8+ cell ratio is usually related to acute viral diseases [120].

Several studies that assessed similar outcomes did not report significant effects from probiotic supplementation compared to placebo. For example, a 12-week study on 141 non-elite marathon runners during pollen season supplementing daily with *L. rhammnosus* GG (LGG) did not find a significant effect on allergic markers [54] or on the incidence of UTRI episodes [55]. Similarly, there was no significant effect on URTI incidence in a study investigating the effect of *L. casei* supplementation in French soldiers participating in intense military training for 3 weeks in a 5-day combat course [56]. In addition, there was no difference in salivary IgA or total and differential leukocyte and lymphocyte subsets.

Gleeson et al. [64] examined the effects of daily supplementation of *L. salivarius* on 66 endurance-based recreational athletes during a four-month period in the spring. There was little effect on frequency, severity or duration of URTIs. In addition, circulating and salivary immune markers did not change over the course of the study and were not different between probiotic and placebo groups. Gleeson et al. [80] also assessed the effect of *L. casei* Shirota on the incidence of URTIs over a 20-week period during the winter in 243 college endurance athletes. Similarly, there was no significant difference between those that consumed the probiotic and the placebo treatment. However, there was a reduction in plasma cytomegalovirus and Epstein Barr virus antibody titers in seropositive athletes compared to placebo, an effect interpreted as a benefit to overall immune status.

While these null findings are important to consider, the current overall body of evidence is weighted notably in favor of probiotics on reduction of URTIs and related symptoms. However, a central issue in relation to the effects of probiotics on immunity, and probiotic research in general, is the large assortment of strains used. Shared, core mechanisms for probiotic function are evident, although some mechanisms may be more narrowly distributed, including those related to immunomodulation [121]. In addition, it is important to note that immune response is complex, as are many of the methodologies used to measure it. For example, an immunomodulatory effect of probiotics is attributed to the release of a large number of cytokines and chemokines from immune cells, which can further impact the innate and adaptive immune systems [122]. Therefore, it is not surprising that the beneficial effect of probiotic administration on the incidence of respiratory illness is possibly linked enhancement of systemic and mucosal immunity. It is possible changes occurred at this level and were not detected in studies that only measured URTI associated metrics. Future work in this area should pair the investigation of URTI incidence and symptomology with other markers of immune response to provide a more thorough understanding of how different probiotics might influence the immune system.

Although less common than symptom outcomes, several studies have provided encouraging evidence in regard to changes in circulating and salivary immune markers. For instance, Clancy et al. [53] sought to determine if immune variables differed between healthy and fatigued recreational athletes after *Lactobacillus* intervention. One month of daily *L. acidophilus* supplementation significantly increased secretion of interferon (IFN)-γ from T cells in fatigued athletes to levels found in healthy athletes and increased the concentration of IFN-γ in saliva of healthy control athletes. IFN-γ is a cytokine intricately linked to mechanisms of control of both virus shedding and disease re-activation. Sashihara et al. [67] evaluated the immunopotentiation and fatigue-alleviation effects of *L. gasseri* OLL2809 supplementation for 4-weeks in 44 university-student athletes. Before and after the treatment period, the subjects performed strenuous cycle ergometer exercise for 1 h. The probiotic supplementation prevented reduced NK cell activity after strenuous exercise which may enhance resistance against infections. In another short-term study, Aghaee et al. [70] reported that a probiotic supplement for 30 days in 16 male athletes increased blood monocyte levels following exhaustive exercise in comparison to placebo control. In a longer duration study, Michalickova et al. [79] investigated the effects of *L. helveticus* Lafti L10 supplementation on systemic humoral and mucosal immune response in 30 elite athletes with a high training load (> 11 h per week) over 14 weeks in the winter. Those that consumed the probiotic exhibited attenuated decreases in total salivary IgA level compared to athletes in the placebo group. Given the fact that mucosal surface is the first-line-of-defense against different pathogens, this finding might have a practical application in terms of prevention of URTIs during strenuous exercise in elite athletes. In comparison to some of the previous studies that didn’t report changes in immune parameters, yet noted a difference in URTI incidence, it is possible that in these circumstances these strains could have displayed antagonistic activities against pathogens and not direct stimulation of the immune system. These effects could include the production of antimicrobials, such as bacteriocins, and low molecular weight compounds such as hydrogen peroxide, lactic acid, and acetic acid [123–125]. These substances could function to outcompete pathogenic bacteria and help in easing or preventing URTI symptoms [126].

In contrast, West et al. [66] did not find significant effects of a synbiotic product including multi-strain probiotics (*Lactobacillus paracasei ssp. paracasei* (*L. casei* 431®), *B. animalis* ssp. *lactis* (BB-12®), *L. acidophilus* LA-5, *L. rhamnosus* GG) on markers of circulating and mucosal immunity in 22 recreational cyclists over a three-week training period. In another small study of the effects of a multi-strain probiotic (*L. acidophilus*, *L. delbrueckii* ssp. *bulgaricus*, *Lactococcus lactis* ssp. *lactis*, *L. casei*, *L. helveticus*, *L. plantarum*, *L. rhamnosus*, *L. salivarius* ssp. *salivarius*, *B. breve*, *Bifidobacterium bifidum*, *B. infantis*, *Bifidobacterium longum*, *B. subtilis*, and *S. thermophilus*) on mucosal immunity, Muhamad & Gleeson [73] did not report a significant alteration in salivary antimicrobial proteins at rest or in response to an acute bout of prolonged exercise in 11 active, healthy adults after 30 days of supplementation. Using a high-dose probiotic treatment, Gill et al. [75] studied 8 male endurance runners who consumed 10 × 10^10^ CFU of *L. casei* for 7 days prior to a two-hour running exercise at 60% VO_2_max in hot ambient conditions (34.0 °C and 32% relative humidity). Supplementation did not enhance salivary antimicrobial proteins responses and subsequent oral-respiratory mucosal immune status above placebo. Finally, Carbuhn et al. [86] explored the effects of *B. longum* 35,624 supplementation in 20 female Division I collegiate swimmers during a 6-week intense training phase on IgA. There were no difference in salivary IgA between groups throughout the study in agreement with a study investigating *B. subtilis* DE111 in collegiate baseball players [83].

Overall, the effect of probiotic supplementation on the immune system in athletes is likely positive and beneficial. Episodes of illness often occur during heavy exercise training periods, a time when athletes obtain the greatest improvements in fitness. Illness that interrupts individual training sessions may prevent athletes from maximizing the effects of their training program. Therefore, probiotic supplementation may be viewed as a viable dietary supplement to support immune function during these periods.

**Table:** 

**Key Points 3 – Effects of Probiotic Supplementation on Immune Function**	
• Athletes may compromise their immune status with high training loads (over-reaching, over-training) which can increase the risk of illness such as URTIs.	
• Overall, the current body of evidence indicates small variable benefits of probiotics during intense training, particularly in endurance athletes, the cohort where the majority of studies are conducted.	
• There is more evidence for the clinical effects of probiotics reducing the incidence URTI and related illness.	
• Positive changes in circulating and salivary immune markers have been more variable and require further research to define more clearly.	

#### The effect of probiotic supplementation on GI tract health

GI problems often occur in endurance athletes and particularly during prolonged events such as cycling, triathlons and marathons [41, 127]. Symptoms such as nausea, cramping, bloating, and diarrhea most likely reflect redistribution of blood flow from the gut to the skin for cooling purposes. Exercise-induced redistribution of blood can result in splanchnic hypoperfusion as a possible mechanism for gut dysfunction [128, 129]. The physical up-and down movement of the gut during running could also explain an increase in the frequency of gut symptoms [41]. Interactions between prolonged exercise, challenging environmental conditions (temperature, altitude, humidity, etc.), and nutrient and fluid intake may also increase risk of gut problems [130]. Disruption in the GI system can impair the delivery of nutrients, and cause GI symptoms and decreased performance. The GI tract and particularly the gut are quite adaptable and can be targeted to improve the delivery of nutrients during exercise while at the same time alleviating some (or all) of the symptoms [131]. A major limitation of studies in this field is that the prevalence of GI illnesses overall is quite low, which makes it difficult to study without a large number of subjects. Probiotic supplementation in combination with other dietary strategies (e.g. consuming well-tolerated foods and drinks, avoiding spicy foods) could assist athletes with a history of GI problems. Moreover, probiotic supplementation potentially could improve GI health which has several indirect athletic benefits. Of the ten studies that assessed GI benefit in athletes and physically active individuals, the majority reported no effect. However, the methodology varied considerably, including probiotic type (species/strain), dosing, duration and study participants, making comparison difficult. Further, the overall result is not conclusive as four studies reported positive results. This latter group included significantly decreased concentrations of zonulin [63] and endotoxin [77], as well as intestinal hyperpermeability [132] and duration of GI-symptom episode. Research in this area has only been conducted intermittently over the past 10 years, with the need for future studies apparent.

In the first reported study investigating the effects of probiotics on GI health, Kekkonen et al. [55], reported no effect of *L. rhamnosus* GG on GI-symptom episodes in marathon runners after a three-month training period. However, the duration of a GI symptom episode was 57% shorter in the probiotic group than in the placebo group. Eight weeks of supplementation with a multi-strain probiotic yogurt in adolescent female endurance swimmers did not affect GI symptoms [44]. In a study of elite union rugby players, subjects given a multi-strain probiotic over 4 weeks did not experience a significant reduction in GI episodes (including nausea, vomiting, diarrhea) compared to the placebo [69].

Investigating markers of gut permeability, West et al. [66] found no significant effect of multi-strain probiotic supplementation on the lactulose/mannitol ratio in active individuals after 3 weeks. Lamprecht et al. [63] explored the effects of 14 weeks of multi-species probiotic supplementation on zonulin from feces in trained men. Zonulin concentrations decreased significantly from slightly above normal into the physiological range in subjects that supplemented with the probiotics. Zonulin is a protein of the haptoglobin family released from liver and intestinal epithelial cells and has been described as the main physiological modulator of intercellular tight junctions [133]. Increased zonulin concentrations are related to changes in tight junction competency and increased GI permeability [133]. The “leak” in the paracellular absorption route enables antigens to pass from the intestinal environment, challenging the immune system to produce an immune response and subsequent inflammation and oxidative stress [134–136]. Lamprecht et al. [63] suggested that the supplemented probiotics may activate the TLR2 signaling pathway resulting in improved intestinal barrier function, thus reducing an athlete’s susceptibility to endotoxemia and associated cytokine production [137].

Shing et al. [46] tested the effects of 4 weeks of multi-strain probiotics supplementation on GI permeability when exercising in the heat in a small group of male runners. To assess GI permeability, subjects ingested lactulose and rhamnose before exercise and post-exercise urine was collected to measure the ratio. Further, urinary claudin-3, a surrogate marker of gut barrier disruption, and serum lipopolysaccharide (LPS) were measured. There was no significant effect on lactulose:rhamnose ratio, urinary claudin-3 or serum LPS and it is possible that 4 weeks may not have been sufficient to detect changes. In short-term, high dose single-strain probiotic supplementation (*L. casei*), male runners under heat stress did not exhibit any marked changes in resting circulatory endotoxin concentration or plasma cytokine profile compared with placebo [76]. Conversely, Roberts et al. [77] reported 12 weeks of supplementation with a multi-strain probiotic/prebiotic significantly reduced endotoxin levels in novice distance triathletes. However, no difference was identified in the assessment of intestinal permeability from urinary lactulose:mannitol ratio. This effect was reported both pre-race and 6 days post-race. Additionally, seven highly-trained endurance athletes who received 4 weeks of *L. salivarius* (UCC118) attenuated exercise-induced intestinal hyperpermeability [132]. Most recently, 12 weeks of probiotic supplementation (*B. subtilis* DE111) had no effect on gut permeability as measured by zonulin in Division I baseball players [83].

**Table:** 

**Key Points 4 – Probiotic Supplementation and Gastrointestinal Health.**	
• GI problems often occur in endurance athletes and can impair the delivery of nutrients, cause GI symptoms and decrease performance.	
• A small number of studies assessing GI benefit in athletes and physically active individuals have yielded mixed results with considerable variation in methodology, making comparison difficult.	
• Positive results reported included decreases in concentrations of zonulin and endotoxin, intestinal hyperpermeability and duration of GI-symptom episodes.	

### Mechanism of action

Given that different strains and product formulations exist, explaining the mechanism of action becomes a rather complex task. An additional challenge in probiotic research is that a mechanism of action involving the gut microbiota is not confirmed, or even examined, in the majority of cases and there certainly are mechanisms outside of the GI tract systemically and in other microbiota niches. Clinical studies track probiotic “inputs” (whether a single strain or multiple strains) and health “outputs”, often without knowing what happens in between. This shortcoming further emphasizes the need to not use the general term probiotics, when describing mechanisms of action, but try to specify the strains [138]. This does not mean the mechanisms are the same for each strain, nor that precise mechanisms have been proven. For example, bacterial strains such as *L. reuteri* SD2112 (ATCC 55730) and *L. reuteri* RC-14 are different genetically and functionally, with the former producing reuterin believed to be important for inhibition of pathogens in the gut [139] and the latter producing biosurfactants that inhibit attachment of uropathogens [140]. Finally, several food products and dietary supplements may contain multiple species and strains in the same product. To fully explain the in-depth mechanisms of action is both out of the scope of this Position Statement and poorly understood in general. However, interested readers are directed to other resources [138, 141]. The question whether multi-strain or multi-species probiotics are better than single strain or single species probiotics depends on the outcome measure, dosage, and study population. Potential additive or even synergistic benefits would need to be validated in a control clinical study, and currently those data do not exist. Mechanisms of action in relation to the effects of probiotic supplementation in athletes has been less described [40]. Here we discuss support of the gut epithelial barrier, increased adhesion to intestinal mucosa, the effects of postbiotics, modulation of the immune system, and improved nutrient absorption.

#### Support of the gut epithelial barrier

The intestinal barrier is a major defense mechanism used to maintain epithelial integrity and protect the host from the environment. Defenses of the intestinal barrier consist of the mucous layer, antimicrobial peptides, secretory IgA and the epithelial junction adhesion complex [142]. Once this barrier function is disrupted, bacterial and food antigens can reach the submucosa and induce inflammatory responses [143, 144]. Consumption of non-pathogenic bacteria can contribute to intestinal barrier function, and probiotic bacteria have been extensively studied for their involvement in the maintenance of this barrier. However, the mechanisms by which probiotics enhance intestinal barrier function are not fully understood. Anderson et al. [145] indicated that enhancing the expression of genes involved in tight junction signaling is a possible mechanism to reinforce intestinal barrier integrity. Probiotics may promote mucous secretion as one mechanism to improve barrier function and the exclusion of pathogens. Several *Lactobacillus* species have been noted to increase mucin expression in human intestinal cell lines and, in the case of a damaged mucosa, may thus help restoration of the mucus layer. However, this protective effect is dependent on *Lactobacillus* adhesion to the cell monolayer, which likely does not occur in vivo [146, 147]. Therefore, mucous production may be increased by probiotics in vivo, but further studies are needed to make a conclusive statement.

Strenuous and prolonged exercise place stresses on the GI tract that increase the likelihood of discomfort, abdominal cramping, acid reflux (heartburn), nausea, vomiting, diarrhea, and permeability of the gut that may allow endotoxemia to occur [41]. Splanchnic hypoperfusion leading to ischemia in the gut is accepted as a principal cause, with additional contributions from nutritional, mechanical (e.g., jarring), and genetic influences that make some individuals more susceptible than others [41]. Probiotic support to increase resilience of the GI tract against ischemia is of interest to athletes, particularly for those involved in prolonged endurance events that have the greatest occurrence of GI problems that can impair or stop performance. Mechanistically, prolonged or strenuous exercise may increase key phosphorylation enzymes [148], disrupting tight junction proteins claudin (influenced by protein kinase A) and occludin (influenced by both protein kinase C and tyrosine kinase). Acute changes in tight junction permeability and paracellular transport may lead to a greater prevalence of systemic LPS. LPS from Gram-negative intestinal bacteria may provoke immune responses and endotoxin-associated symptoms characteristic of GI complaints often experienced in runners [148]. Despite this, research is relatively sparse on whether prolonged training or ultra-endurance events actually result in elevated LPS, particularly in more “recreationally active” athletes; or whether targeted nutrition strategies offer beneficial support. LPS translocation across the GI tract can provoke systemic immune reactions with varied consequences [149]. Specifically, LPS attachment to LPS-binding protein and its transference to an MD 2/TLR4/CD14 complex activates NF-κB and various inflammatory modulators (TNF-α, IL-1β, IL-6 and CRP). This sequence is considered a protective mechanism to minimize bacterial entry across the GI tract. Under normal physiological conditions, endotoxins from gram negative bacteria are usually contained locally, with only relatively small quantities entering the systemic circulation. However, when GI defenses are either disrupted (i.e., luminal damage from exercise) or LPS “sensing” is “overloaded”, a heightened inflammatory response may result which could, in part, relate to GI symptoms associated with exercise [150]. This effect could have implications for daily recovery strategies throughout prolonged training periods, and in the days following ultra-endurance events.

Roberts et al. [77] suggested a multi-strain pro/prebiotic intervention maintains tight junction stability. Further, studies have demonstrated that regular use of probiotics can improve epithelial resistance by establishing competitive “biofilm” formation. Indeed, as LPS types vary across Gram-negative bacteria species, some LPS are poorly sensed by TLR4 and may have more direct impact on NF-κB activation [151]. Therefore, prevention of LPS translocation through maintained epithelial integrity and/or increased preponderance of Gram-positive genera may offer potential therapeutic benefit [152]. Specifically, the provision of bacteria belonging to the *Lactobacillus* genus may work by activating TLR2 and hence produce more favorable innate immune responses [153, 154]. Supplementation with a multi-strain probiotic for 14 weeks decreased fecal zonulin levels, supporting improved tight junction stability through improved intestinal barrier integrity [63]. A mechanistic explanation for an improved intestinal barrier function after probiotic treatment is provided by Karczewski et al. [155], who postulate that certain lactic bacteria might activate the TLR2 signaling pathway. TLR2 is localized in the membranes of intestinal wall cells and from there communicates with microbial products from Gram-positive bacteria [115]. Furthermore, activation of the TLR2 signaling pathway can enhance epithelial resistance in vitro [156]. Therefore, supplemented probiotics may suppress bacteria that activate the zonulin system (e.g. Gram-negative bacteria), settle in the deep intestine, and activate the TLR2 signaling pathway.

#### Adhesion to intestinal mucosa

“Competitive exclusion” is a term used to describe the vigorous competition of one species of bacteria for receptor sites in the intestinal tract over another species. The mechanisms used by one species of bacteria to exclude or reduce the growth of another species include: creation of a hostile microecology, elimination of available bacterial receptor sites, production and secretion of antimicrobial substances and selective metabolites, and competitive depletion of essential nutrients [141]. Adhesion of probiotics to the intestinal mucosa has been shown to favorably modulate the immune system [157, 158] and pathogen antagonism [159]. In addition, probiotics are able to initiate qualitative alterations in intestinal mucins that prevent pathogen binding [160] while some probiotic strains can also induce the release of small peptides or proteins (i.e., defensins) from epithelial cells [161]. These small peptides/proteins are active against bacteria, fungi and viruses [162] and may stabilize the gut barrier function [163]. Specific adhesiveness properties related to the interaction between surface proteins and mucins may inhibit the colonization of pathogenic bacteria and are a result of antagonistic activity by some strains of probiotics against adhesion of GI pathogens [164]. For example, lactobacilli and bifidobacteria can inhibit a broad range of pathogens, including *E. coli*, Salmonella, *Helicobacter* pylori, *Listeria monocytogenes*, and Rotavirus [165–171]. To gain a competitive advantage, bacteria can also modify their environment to make it less suitable for their competitors, such as producing antimicrobial substances (i.e., lactic and acetic acid) [172]. Some lactobacilli and bifidobacteria share carbohydrate-binding specificities with certain enteropathogens [173, 174], which makes it possible for the strains to compete with specific pathogens for the receptor sites on host cells [175]. In general, probiotic strains are able to inhibit the attachment of pathogenic bacteria by means of steric hindrance at enterocyte pathogen receptors [176].

#### Postbiotics

Postbiotics comprise metabolites and/or cell-wall components released by probiotics and offer physiological benefits to the host by providing additional bioactivity [4]. The potential benefits of these metabolites and/or cell wall components should not only be considered to be associated with probiotics but more generally to metabolites produced by bacteria during fermentation, including bile acid fermentation. Several compounds have been collected from several bacteria strains including SCFAs, enzymes, peptides, teichoic acids, peptidoglycan-derived muropeptides, endo- and exo-polysaccharides, cell surface proteins, vitamins, plasmalogens, and organic acids [177–179]. Despite the fact that the mechanisms implicated in the beneficial health effects of postbiotics are not fully elucidated, they possess different functional properties including, but not limited to, antimicrobial, antioxidant, and immune modulation [4]. These properties can positively affect the microbiota homeostasis and/or the host metabolic and signaling pathways, physiological, immunological, neuro-hormone biological, regulatory and metabolic reactions [180, 181].

In the majority of cases, postbiotics are derived from *Lactobacillus* and *Bifidobacterium* species; however, *Streptococcus* and *Faecalibacterium* species have also been reported as a source of postbiotics [177, 179]. SCFAs produced by the gut microbiota act as signaling molecules improving regulation of lipid metabolism, glucose homeostasis, and insulin sensitivity through the activation of receptors such as G protein-coupled receptors (GPRs) to regulate of energy balance while maintaining metabolic homoeostasis [182, 183]. Specific SCFAs (e.g. butyrate, acetate and propionate) also contribute to plasma cholesterol homeostasis in rodents and humans [184]. Some studies [185–187] determined that cell-free extracts from lactic acid bacteria exhibit higher antioxidant capacity than whole cell cultures, suggesting that the antioxidant capacity could be attributed to both enzymatic and non-enzymatic intracellular antioxidants.

Through postbiotic action, it seems plausible that probiotics can increase exercise performance as seen through a delay in fatigue in athletes by virtue of their production of SCFAs. In addition, species within the *Lactobacillus* genus synthesize lactic acid, which is converted to butyrate and later to acetyl-CoA, which is used in the Krebs Cycle to generate adenosine triphosphate (ATP). However, these processes occur mostly in the gut so whether or not this would impact skeletal muscle performance remains to be determined [188]. Another mechanism is by antioxidant action, which can attenuate muscle injury induced by reactive oxygen species, among others [92]. Antioxidant effects found in probiotics are linked to the synthesis of antioxidant substances such as vitamins B1, B5 and B6 [141]. Moreover, probiotic supplementation reduces the risk of developing hyperglycemia, a condition known to be linked to oxidative stress [189, 190]. Finally, the improvement in intestinal homeostasis, including the absorption process, may favor the absorption of antioxidants, increasing the availability of these substances [58].

One of the proposed mechanisms involved in the health benefits afforded by probiotics includes the formation of low molecular weight compounds (< 1000 Da), such as organic acids, and the production of antibacterial substances termed bacteriocins (> 1000 Da). Organic acids, in particular acetic acid and lactic acid, have a strong inhibitory effect against Gram-negative bacteria, and are considered the main antimicrobial compounds responsible for the inhibitory activity of probiotics against pathogens [191–193]. The undissociated form of the organic acid enters the bacterial cell and dissociates inside its cytoplasm. The eventual lowering of the intracellular pH or the intracellular accumulation of the ionized form of the organic acid can lead to the death of the pathogen [194].

Intestinal bacteria also produce a diverse array of health-promoting fatty acids. Certain strains of intestinal bifidobacteria and lactobacilli can produce conjugated linoleic acid (CLA), a potent anti-carcinogenic agent [195, 196]. An anti-obesity effect of CLA-producing *L. plantarum* has been observed in diet-induced obesity in mice [197]. Recently, the ability to modulate the fatty acid composition of the liver and adipose tissue of the host upon oral administration of CLA-producing bifidobacteria and lactobacilli has been demonstrated in a murine model [196]. Finally, certain probiotic bacteria are able to produce so-called de-conjugated bile acids, which are derivatives of bile salts. De-conjugated bile acids show a stronger antimicrobial activity compared to that of the bile salts synthesized by the host organism [141].

#### Modulation of the immune system

Numerous studies have shown that prolonged intense physical exercise is associated with a transient depression of immune function in athletes. While moderate exercise beneficially influences the immune system [198], a heavy schedule of training and competition can impair immunity and increase the risk of URTIs due to altered immune function [116, 199, 200]. Both innate immunity and acquired immunity are decreased following prolonged exercise [199–201]. It is well known that probiotic bacteria can exert an immunomodulatory effect; however, research from non-athletic populations may not be translatable to athletes. Further, the manipulation and control of the immune system by probiotics is difficult to evaluate and make general conclusions. However, several studies investigating the effects of probiotics in athletes have reported improvement in low-grade inflammation [42, 63], as well as increased resistance to URTIs [57, 60, 69, 78] and reduced duration of URTI [79].

Modulation of the immune system to increase defenses against URTIs currently is the most extensively researched area. The GI tract is a major gateway for pathogen entry, and as such, is heavily protected by the immune system. The immune system can be divided between the innate and adaptive systems. The adaptive (acquired) immune response depends on B and T lymphocytes, which are specific for particular antigens. In contrast, the innate immune system responds to common structures called pathogen-associated molecular patterns (PAMPs) shared by the vast majority of pathogens [202]. The primary response to pathogens is triggered by pattern recognition receptors (PRRs), which bind PAMPs. The best-studied PPRs are TLRs. In addition, extracellular C-type lectin receptors (CLRs) and intracellular nucleotide-binding oligomerization domain-containing protein NOD-like receptors are known to transmit signals upon interaction with bacteria [203]. It is well established that probiotics can suppress intestinal inflammation via the downregulation of TLR expression, secretion of metabolites that may inhibit TNF-α from entering blood mononuclear cells, and inhibition of NF-ĸB signaling in enterocytes [202].

Probiotics can enhance innate immunity (first-line-of-defense) by upregulating immunoglobulins, antimicrobial proteins, phagocytic activity, and natural killer cell activity, and enhance acquired immunity by improving antigen presentation and function of T and B lymphocytes to neutralize pathogens and virally-infected cells [10, 204]. These effects are of particular importance to athletes because exercise may increase susceptibility to URTIs by decreasing salivary IgA, decreasing cell-mediated immunity by decreasing type 1 T lymphocytes to make recurrent infections more likely, and increasing glucocorticoid suppression of monocyte/macrophage antigen presentation and T lymphocyte functions [205, 206]. The majority of placebo-controlled clinical trials assessing the efficacy of probiotics for reducing incidence, duration, and severity of URTI in athletes report beneficial outcomes. However, many different probiotics have been used and the differences in trial protocols and outcome measures complicate the drawing of more specific conclusions.

#### Improved nutrient absorption

Supplementation with some probiotic strains has been suggested to improve dietary protein absorption and utilization [207]. While not fully elucidated, several studies indicate a plausible role [208], yet a clear mechanism of action is lacking. As noted, probiotics can potentially improve intestinal barrier function by modulating tight junction permeability which may improve nutrient absorption.

Improving the digestibility of protein can speed recovery of strength after muscle-damaging exercise [209], and promote glycogen replenishment after exercise. *B. coagulans* produce digestive enzymes [97] active under gut conditions (alkaline proteases). These proteases can digest proteins more efficiently than the endogenous human proteases alone [96]. *B. coagulans* GBI-30, 6086 enhances the health of the cells of the gut lining improving nutrient absorption including minerals, peptides, and amino acids by decreasing inflammation and encouraging optimum development of the absorptive area of the villi [98].

In a computer-controlled in vitro model of the small intestine, *B. coagulans* GBI-30, 6086 enhanced amino acid absorption while improving colon health [208]. In recreationally-trained males, Jäger et al. [43] found the co-administration of *B. coagulans* GBI-30, 6086 and 20 g of protein improved recovery 24 and 72 h, and muscle soreness 72 h post-exercise. Furthermore, Toohey et al. [103], noted *B. subtilis* DE111 supplementation with a post-workout recovery drink containing 20 g of protein reduced body fat percentage after 10 weeks of resistance training compared with the same post-workout recovery drink and a placebo in female athletes. Toohey et al. [103] speculated improved amino acid uptake in the probiotic group may have resulted from more efficient protein digestion, simulating the effects of a higher daily protein intake.

**Table:** 

**Key Points 5 – Mechanisms of Action**	
• There are dozens of bacterial strains that can be considered as probiotics, particularly those that produce lactic acid. However, each strain is unique with respect to how it responds to and affects the host.	
• The mechanisms underlying the beneficial effects of probiotics in athletes are largely unknown but are likely to be multifactorial.	
• Consumption of some probiotic strains may improve intestinal barrier function by modulating tight junction permeability. However, the mechanisms by which probiotics enhance intestinal barrier function are not sufficiently studied.	
• Adhesion of probiotics to the intestinal mucosa may be a mechanism for modulation of the immune system. Probiotics also cause alterations in intestinal mucins that prevent pathogen binding.	
• Probiotics may support microbiota and postbiotic production which possess different functional properties including, but not limited to, antimicrobial, antioxidant, and immunomodulatory.	
• Probiotics may enhance innate immunity by upregulating immunoglobulins, antimicrobial proteins, phagocytic activity, and natural killer cell activity, and also enhance acquired immunity by improving antigen presentation and function of T and B lymphocytes to neutralize pathogens and virally-infected cells.	
• Probiotics can potentially modulate intestinal permeability and health of the cells of the gut lining improving nutrient absorption including minerals, peptides, and amino acids by decreasing inflammation and encouraging optimum development of the absorptive area of the villi.	

### Safety and health

The concept of probiotics is not new. Around 1900 Nobel laureate, Elie Metchnikoff, discovered that the consumption of live bacteria (*L. bulgaricus*) in yogurt or fermented milk improved some biological features of the GI tract [210]. Bacteria with claimed probiotic properties are now widely available in the form of foods such as dairy products and juices, and also as capsules, drops, and powders. Probiotics have been used safely in foods and dairy products for over a hundred years. Some of the most common commercially available strains belong to the *Lactobacillus* and *Bifidobacterium* genera. In this respect, well-studied probiotic species include *Bifidobacterium* (ssp. *adolescentis, animalis, bifidum, breve, and longum*) and *Lactobacillus* (ssp. *acidophilus, casei, fermentum, gasseri, johnsonii, reuteri, paracasei, plantarum, rhamnosus*, and *salivarius*) [211]. An international consensus statement in 2014 indicated that these are likely to provide general health benefits such as normalization of disturbed gut microbiota, regulation of intestinal transit, competitive exclusion of pathogens, and production of SCFAs [1].

Beyond athletes and physically active individuals, there is a large body of preclinical and clinical research on the GI benefits of probiotics in healthy individuals and in a wide range of health conditions. These applications include treatment and prevention of acute diarrhea, prevention of antibiotic-associated diarrhea, treatment of hepatic encephalopathy, symptomatic relief in irritable bowel syndrome, and prevention of necrotizing enterocolitis in preterm infants [212]. Overall, probiotics have an excellent safety profile with a large majority of clinical trials involving probiotics not giving rise to major safety concerns [213]. Of the adverse events (AEs) commonly reported, Marteau [214] outlined four classes of possible side effects of probiotic use: systemic infections, detrimental metabolic effects, cytokine-mediated immunologic adverse events in susceptible individuals, and transfer of antibiotic resistance genes. Of these, particular concern relates to probiotics potential to create (not improve or treat) systemic infections [49, 64, 215]. Further, probiotics have been studied in vulnerable groups, including infants, patients with severe acute pancreatitis, inflammatory bowel diseases, liver diseases, HIV, and other conditions [213, 216–218] with even greater cause for concern with the small number of products that contain high concentrations of up to 450–900 billion live bacteria per dose [211]. Many of the studies reporting AEs (rarely serious AEs) either do not utilize the appropriate biological sampling and identification techniques or AEs are poorly reported.

Commercially available probiotic products can be divided into single-strain (defined as containing one strain of a well-defined microbial species) and multi-strain (containing more than one strain of the same species or genus). The term multispecies is also used for products that contain strains from more than one genus [211], for example a product with a *L. acidophilus* strain, a *L. reuteri* strain, and a *B. longum* strain. Treatment with probiotics may involve the consumption of large quantities of bacteria, so safety is a primary concern. There are two aspects to safety: establishing the adverse effect profile of specific single-strain and multi-strain supplements (i.e., the safety of the strain(s) per se), and ensuring that marketed supplements meet stringent quality standards to ensure the correct strains are present and the product is free of contamination [217].

Safety assessments should take into account the nature of the specific probiotic microbe, method of administration, level of exposure, health status of the recipients, and the physiological functions the microbes are intended to perform [213]. However, most probiotics in commercial use are derived from fermented foods with a long history of safe consumption, or from microbes that may colonize healthy humans [212]. All common probiotic species are considered safe for the general population by the European Food Safety Authority (EFSA), although this definition does not provide guidance on the increasing use of probiotics in people with medical conditions. Moreover the benefits of probiotics are not validated by EFSA, jeopardizing the use of the term probiotic without an approved claim with some exceptions such as in Italy, Czech Republic, and Bulgaria [211]. Going beyond history of safe use, since 2007 the EFSA lists species presumed safe for human consumption under the “Qualified Presumption of Safety” (QPS) concept. The approach is based on experience that for selected organisms there are no reasonable safety concerns for human health. The list regularly monitors the body of knowledge through extensive scientific literature review, applied to a wide array of micro-organisms added in the food-chain. The QPS list concerns consumption by the general healthy population and does not take into consideration potential risks for vulnerable populations and this is clearly mentioned. The U.S. Food and Drug Administration (FDA) classifies probiotics individually but has classified many as Generally Recognized As Safe (GRAS), safe for the use in foods and infant products [219].

A systematic literature review of probiotic safety published in 2014 reported that “the overwhelming existing evidence suggests that probiotics are safe” for the general population, and that critically ill patients, postoperative and hospitalized patients and immunocompromised patients were the most at-risk groups wherein AEs occurred [220]. The general consensus is that probiotic ingestion is safe [221, 222], with large doses well tolerated and failing to exhibit any toxicity [223]. Indeed, low CFU dosage and intervention periods between 2 weeks to 6 months are generally used within clinical research models [224, 225]. In this position stand, which reviews studies focused on probiotic supplementation in athletes and physically active individuals, 11 studies measured AEs and general supplementation tolerance, while 30 studies did not. Of the 11 studies, a general consensus was made to conclude that probiotic supplementation was generally well tolerated with a very low level of adverse health effects. There was one instance in which mild GI symptoms (5 episodes) were reported, including flatulence and stomach rumbles during supplementation with a multi-strain probiotic in 22 active individuals [66]. AEs are often not well recorded in nutritional studies in general and probiotics are no exception to this. Overall, from the current body of research probiotic supplementation for healthy athletes and physically active individuals appears safe. Caution is warranted for those with serious health conditions, such as severe acute pancreatitis, inflammatory bowel diseases, liver diseases, and HIV. In these instances, it is advised that the patient consult with their health care practitioner before supplementing. Another consideration is supplementing evidence-based dosages and keeping the probiotic properly stored. Unlike, other familiar sports supplements, probiotics are live organisms and may require specific storage requirements including refrigeration.

**Table:** 

**Key Points 6 – Safety and Health.**	
• Probiotics have been used safely in foods and dairy products for over a hundred years.	
• Well-studied probiotic species *include Bifidobacterium (ssp. adolescentis, animalis, bifidum, breve, and longum) and Lactobacillus (ssp. acidophilus, casei, fermentum, gasseri, johnsonii, reuteri, paracasei, plantarum, rhamnosus, and salivarius).*	
• Safety assessments should take into account the nature of the probiotic microbe, method of administration, level of exposure, health status of the recipients, and the underlying physiological functions the microbes are intended to perform.	
• Four classes of possible side effects are commonly reported from probiotic use in vulnerable patient groups: systemic infections, detrimental metabolic effects, cytokine-mediated immunologic adverse events in susceptible individuals, and transfer of antibiotic resistance genes.	
• The current body of research of probiotic supplementation for healthy athletes and physically active individuals suggests that they are safe for use.	
• Caution is warranted for those with serious health conditions. In these instances, patients should consult with their health care practitioner before supplementing.	
• Consumers are advised to supplement with probiotics strains and products within evidence-based dosages.	

### Regulation

Currently there is no clear set of recommendation or guidelines on probiotic use for athletes. The current body of research has investigated a wide variety of species/strains, duration of use, and dosages with several different intended purposes (Table 4). The effects of probiotics are strain specific, and therefore, strain identity is important to link to a specific health effect as well as to enable accurate surveillance and epidemiological studies. Unfortunately, government regulatory organizations are highly varied across national borders and jurisdictions in regulation of probiotics, making uniform recommendations difficult.

**Table 4:** Dosage range in studies investigating the effect of specific probiotic genera in athletes and physically active individuals

Type	Dosage range
*Lactobacillus* (*n* = 35)	1 × 10^9^ – 10 × 10^10^ CFU
*Bifidobacterium* (*n* = 18)	7 × 10^7^–9.5 × 10^9^ CFU
*Streptococcus* (*n* = 8)	5 × 10^9^–4.5 × 10^10^ CFU
*Bacillus* (*n* = 5)	5 × 10^8^ – 5 × 10^9^ CFU
Multi- species/strain (*n* = 17)	2 × 10^9^–4.5 × 10^10^ CFU

In 2001, the FAO/WHO held the *Expert Consultation on Evaluation of Health and Nutritional Properties of Probiotics*, to develop standardized guidelines for evaluating probiotics in food that could lead to the substantiation of health claims [226]. The proposed guidelines recommend: 1) identifying of the genus and species of the probiotic strain by using a combination of phenotypic and genotypic tests as clinical evidence suggesting that the health benefits of probiotics may be strain specific, 2) in vitro testing to delineate the mechanism of the probiotic effect, and 3) substantiating the clinical health benefit of probiotic agents with human trials. Additionally, safety assessment of the probiotic strain should at a minimum determine: 1) patterns of antimicrobial drug resistance, 2) metabolic activities, 3) side effects noted in humans during clinical trials and after marketing, 4) toxin production and hemolytic potential if the probiotic strain is known to possess those properties, and 5) lack of infectivity in animal studies [226].

The regulation of probiotics differs between countries as there is no universally agreed framework. For the most part, probiotics are categorized as food and dietary supplements because most are delivered by mouth as a food or supplement. For example, Health Canada has provided a Natural Health Product monograph that includes dosage form(s), use(s) or purpose(s) recommended as well as minimum quantities for *L. johnsonii* (La1/Lj1/NCC 533, an adjunct to physician-supervised antibiotic therapy in patients with *H. pylori* infections, 1.25 × 10^8^ CFU) (all strains, 1 × 10^7^ CFU), *L. rhamnosus* (GG, Management of acute infectious diarrhea, 6 × 10^9^ CFU, management/risk reduction of antibiotic-associated diarrhea, 1 × 10^10^ CFU) (all strains, 1 × 10^7^ CFU), and *S. boulardii / S. cerevisiae* (all strains, Risk reduction of antibiotic-associated diarrhea, 1 × 10^10^ CFU) (all strains, 1 × 10^7^ CFU). The probiotic product monograph contains both bacteria and fungi which have been pre-approved for the use or purpose which allows claims; “source of probiotics”, “helps support intestinal/gastrointestinal health”, “could promote a favorable gut flora” with 1 × 10^7^ CFU daily. The minimum daily dose is the sum of CFU per day provided by all live microorganisms that are present in the product, and not the minimum amount of CFU per day for each of the microorganisms. Further, a duration of use statement is not required, nor is there any guidance provided. Cautions include; “If you have fever, vomiting, bloody diarrhea, or severe abdominal pain, consult a health care practitioner prior to use” and “If symptoms of digestive disorders (e.g., diarrhea) occur, worsen and / or persist beyond 3 days, discontinue use and consult a health care practitioner.” [227]. In Canada, probiotics have two modes of sale on the market, Natural and Non-Prescription Health Products Directorate (NNHPD) and Food Directorate [3, 228]. Health Canada uses a pre-market approval process for non-food like applications such as capsules, tablets, softgels and powders which requires companies to acquire a Natural Product Number (NPN) prior to bringing to market [3]. Table 5 below details the current licensed products and claims specific to sport performance using probiotic strain(s) in or outside the pre-approved monograph. This list is open access through the Health Canada LCNHPD (Licensed Natural Health Products Database) which allows consumers and retailers the ability to review claims on packaging to approved claims by the NNHPD [229].

**Table 5:** Approved Canadian Probiotics Claims for Sports Performance

NPN	Probiotic Species Used (Strains if available) and Potency	Sport Specific Claims Supported by Research outside of monograph
80,080,307	*B. breve* BR03 5 Billion CFU *S. salivarius* ssp. *thermophilus* FP4 5 Billion CFU	Helps maintain gastrointestinal health which may assist in normal recovery of performance following exercise.
80,077,863	*B. coagulans* GBI-30, 6086 1 Billion CFU	*B. coagulans* GBI-30, 6086 could be used to improve symptoms of delayed onset muscle soreness (DOMS) after exercise. *B. coagulans* GBI-30, 6086 helps maintain gastrointestinal health which may assist in a normal recovery of performance following exercise.
80,040,732	*L. helveticus* 400 million CFU *B. longum* subsp. *longum* 600 million CFU	Helps maintain the health of the immune system following periods of physical stress.
80,064,384	*L. helveticus* 10 Billion CFU	Promotes gastrointestinal health in physically active adults Helps reduce the incidence of cold-like symptoms in adults with exercise-induced stress
80,064,386	*L. helveticus* 10 Billion CFU × 2	Promotes GI health in physically active adults Helps support immune defenses against winter infections in healthy adults and in those having weakened immunity due to intensive sports activities Promotes GI health, immune health and general well-being in physically active adults (including sporty individuals like athletes) Reduces symptoms with upper respiratory tract infections Helps reduce incidence of cold-like symptoms in adults with exercise-induced stress With 20 Billion CFU per day, this product helps support the first line of body’s immune defenses (IgA production), which may be associated with lowering URTI risk in physically active adults (such as competitive athletes)
80,050,736	*B. animalis* subsp. *lactis* 23 Billion CFU *B. animalis* subsp. *lactis* 50 million CFU *B. animalis* subsp. *lactis* 1 Billion CFU *B. bifidum* 50 million CFU *B. longum* subsp. *infantis* 100 million CFU *L. acidophilus* 24.8 Billion CFU *L. acidophilus* 1 Billion CFU	Reduces the risk of developing upper respiratory tract illness in physically active adults Reduces the duration of URTI in physically active adults
80,064,494	*B. animalis* subsp. *lactis* BI-04 10 Billion CFU *B. animalis* subsp. *lactis* Bi-07 10 Billion CFU *L. acidophilus* NCFM 10 Billion CFU *L. paracasei* LPC-37 10 Billion CFU	Helps reduce the risk of developing URTI in physically active adults
80,068,830	*B. animalis* subsp. *lactis* Bi-04 2 Billion CFU	Reduces the risk of developing URTI in physically active adults Reduces the duration of URTI in physically active adults
80,080,161	*B. longum* subsp. *longum* 320 million CFU *L. helveticus* 2.68 billion CFU *L. helveticus* 5 Biillion CFU	Promotes GI health, immune health and general well-being in physically active adults (including sporty individuals like athletes) Reduces symptoms associated with upper-respiratory tract illness (URTI). Helps shorten the duration of URTI episodes Helps reduce the incidence of cold-like symptoms in adults with exercise-induced stress Helps support the first line of the body’s immune defenses (IgA production), which may be associated with lowering URTI risk in physically active adults (such as competitive athletes) Helps support immune defenses against winter infections in healthy adults and in those having weakened immunity due to intensive sports activities Helps to reduce gastrointestinal discomfort (e.g., abdominal pain, nausea, vomiting) in those experiencing mild to moderate stress resulting from life events (e.g., academic exams) Helps to moderate general feelings of anxiety Promotes a healthy mood balance Helps to reduce stress-related gastrointestinal complications such as abdominal pain
80,089,514	*B. bifidum* 3 Billion CFU *L. helveticus* 5 Billion CFU	Helps support immune defenses against winter infections in healthy adults and in those having weakened immunity due to intensive sports activities Helps to alleviate gastro-intestinal (GI) disturbances like flatulence, constipation, bloating and abdominal cramps in healthy adults Promotes GI health, immune health and general well-being in physically active adults (including sporty individuals like athletes) Reduces symptoms associated with upper-respiratory tract illness (URTI) Helps shorten the duration of URTI episodes Helps reduce the incidence of cold-like symptoms in adults with exercise-induced stress Helps support the first line of the body’s immune defenses (IgA production), which may be associated with lowering URTI risk in physically active adults (such as competitive athletes) Helps reduce the incidence of cold-like symptoms in stressed adults
80,091,068	*B. animalis* subsp. *lactis* 2 Billion CFU *L. acidophilus* 1 Billion CFU *L. acidophilus* 3 Billion CFU *L. plantarum* 14 Billion CFU	Reduces the risk of developing upper respiratory track illness in physically active adults Reduces the duration of upper respiratory tract illness in physically active adults
80,091,070	*B. animalis* subsp. *lactis* 2 Billion *L. acidophilus* 1 Billion *L. acidophilus* 3 Billion *L. plantarum* 14 Billion	Reduces the risk of developing upper respiratory track illness in physically active adults Reduces the duration of upper respiratory tract illness in physically active adults
80,087,974	*B. animalis* subsp. *lactis* 2.81 Billion CFU *B. animalis* subsp. *lactis* 1.47 Billion CFU *B. animalis* subsp. *lactis* 810 million CFU *B. animalis* subsp. *lactis* 530 million CFU *B. bifidum* 28 million CFU D-Glucose 13 mg D-Xylose 13 mg L-Arabinose 7 mg *L. acidophilus* 630 million CFU *L. casei* 610 million CFU *L. paracasei* 690 million CFU *L. plantarum* 890 million CFU *L. salivarius* 560 million CFU Xylooligosaccharides 631 mg	Reduces the risk of developing upper respiratory track illness in physically active adults Reduces the duration of upper respiratory tract illness in physically active adults

Japan is viewed by many to be a global market leader given that probiotics are available as both foods and drugs [230], and was the first global jurisdiction to implement a regulatory system for functional foods and nutraceuticals in 1991. Under Japanese regulations, probiotic products are in a distinct category of foods known as Foods for Specific Health Uses (FOSHU). For probiotic food products, efficacy claims are prohibited on the labeling. If claims are to be made about efficacy, one must obtain special permission from the Ministry of Health and Welfare (MHLW) for the product to be considered FOSHU, for which substantiation of efficacy and safety is a mandatory requirement [231]. In Brazil, probiotics are considered as functional foods, and considered to be different from food. But legislation asks for safety and efficacy demonstration of food products and hence all these products must be registered and approved by a health authority called National Health Surveillance Agency Brazil (ANVISA) [230].

In the European Union, probiotics and food supplements are regulated under the Food Products Directive and Regulation (regulation 178/2002/EC; directive 2000/13/EU). All health claims for probiotics have to be authorized by EFSA which has issued a list of microbial cultures that have a Qualified Presumption of Safety [232], meaning that they do not require safety assessments. The EFSA is also responsible for assessing health claims made for probiotic products. So far, EFSA has rejected all submitted health claims for probiotics. While rigorous scrutiny of product claims is apparent, there appears to be little regulation of the manufacturing process and almost no post-marketing regulatory follow-up [233].

In the United States, government regulation of probiotics is complex. Depending on a probiotic product’s intended use, the FDA might regulate it as a dietary supplement, a food ingredient, or a drug. Many probiotics are sold as dietary supplements, which do not require FDA approval before they are marketed. Dietary supplement labels are permitted to make claims about how the product affects the structure or function of the body without FDA approval, but they cannot make health claims (claims that the product reduces the risk of a disease) without the FDA’s approval [234]. Further, dietary supplements are required to comply with Good Manufacturing Practice guidelines, but these do not extend to testing quality or efficacy [233]. From the examples provided, it is apparent that the current approach to regulation is inadequate and can lead to problems of quality, safety, and claim validity in commercial probiotic products used in a medical context, including those used in vulnerable populations [233].

In January 2017, the Council for Responsible Nutrition (CRN) and the International Probiotics Association (IPA) announced the development of scientifically-based best practices manufacturing guidelines for the labeling, storing, and stability testing of dietary supplements and functional foods containing probiotics [235]. These guidelines were designed to facilitate transparency and consistency in the probiotic sector. A key element of the guidelines is labelling probiotic products in CFU, the scientifically accepted unit of measure for probiotics and used to report probiotic quantity in many studies conducted to assess the safety or benefits of probiotics. Consistent with scientific literature, CFU are commonly used on probiotic product labels in many jurisdictions around the world to help consumers and healthcare professionals identify products providing probiotics in amounts shown to have benefit. However, United States regulations require dietary ingredients (with the exception of some vitamins) be labeled by weight. Labeling probiotic quantity by weight is not meaningful because this measure does not indicate the viability of the microorganisms in the product throughout shelf life. To the contrary, CFU are more representative of the quantity of viable microorganisms and gives consumers and healthcare professionals accurate information. The FDA has recently agreed that in addition to weight, probiotic amounts can also be labelled in CFU.

Upon examining the relevant literature investigating the effects of probiotic supplementation on athletes and those physically active, the genera commonly used included *Lactobacillus* (*n* = 35), *Bifidobacterium* (*n* = 18), *Streptococcus* (*n* = 8) and *Bacillus* (*n* = 5) (Table 3). In addition, several studies used a combination of species and strains (*n* = 17), ranging from two up to 14 different species/strains. The dose of probiotic administered is an important factor to be considered. In two reviews related to dietary supplementation in athletes, dosing regimens were reported in the range between 1 × 10^9^ to 4 × 10^10^ CFU [10, 40]. In a 2018 consensus statement, the International Olympic Committee noted moderate support for probiotic use in athletes with a daily dose of 1 × 10^10^ live bacteria [5]. In our review, we report a wide range of doses (Table 4), and in several studies the dosage was not reported.

Similar to the type of probiotic used, the duration of supplementation has also been variable in the studies reviewed (Table 3). The shortest duration lasted 7 days [75, 76] and the longest lasted 150 days [68]. The duration and consistency of probiotic supplementation are important factors. Coqueiro et al. [188] noted that in clinical practice probiotic supplementation should be implemented for at least 14 days prior to competition or important events for the athlete. Therefore, studies that supplement for a similar or shorter period should be evaluated with caution. With the interruption of probiotic intake, there is a reduction in the microorganism administered in the colon, and with 8 days of supplementation discontinuation, the probiotic is no longer detectable in the gut [236]. Finally, there is some limited evidence that discrepancies exist between males and females, even after supplementation of probiotics with the same dose [61]. Future studies are needed in this area, with the intention of establishing a recommendation for each sex.

**Table:** 

**Key Points 7 Regulation**	
• No universally agreed upon framework exists for regulating commercial products containing probiotics across countries globally.	
• Probiotic products should be labelled in CFU, the scientifically accepted unit of measure for probiotics and used to report probiotic quantity in many studies conducted to assess the safety or benefits of probiotics.	
• Dosing regimens typically fall in range between 1 × 10^9^ to 1 × 10^11^ CFU.	
• The IOC noted moderate support for probiotic use when administered for several weeks in athletes with a daily dose of 1 × 10^10^ CFU.	
• Genera of commonly used probiotics include Lactobacillus (*n* = 35), Bifidobacterium (*n* = 18), Streptococcus (*n* = 8) and Bacillus (*n* = 5).	
• Single-strain and multi- species/strain products are commonly used, but combinations and individual dosing recommendations are not currently understood	
• Males and females may respond to probiotic supplementation differently. Future research is needed in this area.	

### Future directions

Overall, the effects of probiotics in athletes have received less attention compared to animal studies and human clinical conditions in the general population. A PubMed search conducted in October 2019 yielded the following listings for various combinations of key terms: probiotic and athlete, *n* = 145; probiotic and rodent, *n* = 3407; probiotic and diabetes, *n* = 844; probiotic and child, *n* = 2930; probiotic and elderly, *n* = 2257. Clearly, the focus of the research community has been investigating the beneficial effects of probiotics on gut and immune health in various subgroups of the general population. In animals, probiotics have been associated with benefits including normalizing age-related drops in testosterone levels [237], increasing neurotransmitter synthesis [238], reducing stress-induced cortisol levels [239], reducing inflammation [100] and improving mood [240]. However, all these potential benefits lack current substantiation in human intervention trials in an athletic population. Here we discuss future research opportunities to explore in relation to the microbiome and athletes.

#### Body composition and muscle mass

It is well known that to increase levels of muscle mass, resistance training must be included in exercise regimens. Probiotic supplementation, both with and without resistance training, can decrease levels of body weight and fat mass in overweight and obese individuals, as well as female athletes [103, 241, 242]. Increases in fat free mass, however, have only been shown in animal models. Chen and colleagues [92] supplemented male Institute of Cancer Research (ICR) strain mice with *L. plantarum* TWK10 for 6 weeks. Mice were divided into three groups and daily doses of 0, 2.05 × 10^8^, or 1.03 × 10^9^ CFU were given to each group, respectively. The dosages chosen were modified from a comparable human dose equivalent to mouse body size. Relative muscle weight (%), as measured by combining the gastrocnemius and soleus muscles, were significantly increased in mice consuming the probiotic compared to placebo. Additionally, the number of type I fibers were increased significantly.

Mechanistically, it is plausible that *Lactobacillus* strains decrease levels of inflammation, thereby decreasing activation of intracellular proteins linked to muscle atrophy, which may eventually link to an observed increase in muscle mass. Chen et al. [92] also determined that probiotic supplementation increased forelimb grip strength and swim-to-exhaustion performance in mice, which may or may not have been related to improvement in muscle mass. Though improvements in body composition have been shown in humans, more studies examining decreased inflammation as a mechanism to increase muscle mass, in conjunction with reduction in fat mass, is warranted.

#### Buffering capacity in exercising muscles

Physiological fatigue, such as extreme fatigue after exercise, is accompanied by poor athletic performance and loss of favorable working conditions for tissues [243]. In response to higher intensity exercise, the concentration of lactate and hydrogen ions increased markedly resulting in an acidification in muscle and subsequent fatigue [244, 245]. Approximately 75% of the total amount of lactate produced is used for oxidative production of energy in the exercising body and can be utilized for the de novo synthesis of glucose in the liver [246].

Probiotic supplementation may have potential to remove and utilize blood lactate after exercise. For instance, most *Lactobacillus* species produce lactic acid, which could facilitate the production of butyrate by lactate-utilizing bacteria that first produce acetyl-CoA from lactate [247]. In the classical pathway, the enzymes phosphotransbutyrylase and butyrate kinase convert butyryl-CoA to butyrate and coenzyme A with concomitant formation of ATP. Thus, probiotics and the gut microbiota could play important roles in maintaining normal physiology and energy production during exercise. Several animal studies have been conducted with promising results. In mice who consumed a probiotic kefir daily over 4 weeks, swimming time-to-exhaustion was significantly longer, forelimb grip strength was higher and serum lactate, ammonia, blood urea nitrogen (BUN), and creatine kinase levels were lower after the swimming test [248]. In mice supplemented with *L. plantarum* TWK10 over 6 weeks, supplementation dose-dependently increased grip strength and endurance swimming time and decreased levels of serum lactate, ammonia, creatine kinase, and glucose after an acute exercise challenge [92]. Furthermore, the number of type I fibers in gastrocnemius muscle significantly increased with LP10 treatment. In a six-week human double-blind placebo-controlled clinical study, young healthy amateur runners supplemented with *L. plantarum* TWK10 and underwent an exhaustive treadmill exercise measurements and related biochemical indexes [85]. The TWK10 group had significantly higher endurance performance and glucose content in a maximal treadmill running test compared to the placebo group (*P* < 0.05), indicating that TWK10 supplementation may be beneficial to energy harvest. Together, these studies suggest a role in which certain probiotics may enhance energy harvesting, and have health-promotion, performance-improvement, and anti-fatigue effects. These are areas that may warrant further research consideration.

#### Considerations for future study designs

Several important methodological shortcomings in research design should be addressed to improve scientific evidence for the biological and clinical benefits of probiotics. For example, discrepancies between men and women, even after supplementation of probiotics with the same dose, are evident [61]. In this sense, in studies with both sexes, conflicting results may occur. In many instances and products, the recommendation for probiotic supplementation is no different for men and women, necessitating studies investigating this topic, with the intention of establishing a recommendation for each sex.

Other design concerns include the relatively small number of subjects, which may compromise the accuracy and interpretation of results. The period of supplementation is another important factor as the time of adaptation of the organism to the probiotic is approximately 14 days. Thus, studies that supplement for a similar or shorter period should be evaluated with caution. Further, with the interruption of probiotic intake, there is a reduction in the microorganism administered in the colon, and with 8 days of supplementation discontinuation, the probiotic is no longer detectable in the gut [236]. In clinical practice, it is common sense that probiotic supplementation should be implemented for at least 14 days prior to competition or important events for the athlete, given that during this period the GI tract adapts to the administered microorganism [188], and there may be mild, transient GI symptoms, such as flatulence [10]. The long-term effects of probiotic administration in athletes over several months or years on gut health, immune function and rates of illness are unclear, as in most studies the supplementation period was between 4 to 16 weeks.

Since many effects are dose-dependent, the amount of probiotic administered is an important factor to be considered. The range of oral probiotic supplementation is, approximately, 10^8^–10^9^ CFU per day, however, this value varies in each country [249, 250] and notably, no specific probiotic recommendation has been established for athletes or physical activity practitioners. Most of the studies do not control for previous levels of physical activity, so individuals within the same study may have very different levels of physical activity, making comparisons unrealistic. Finally, very few studies have evaluated the performance in strength exercises after supplementation with probiotics and this is an important area of sports and physical training to be studied.

#### Hormonal balance

Oral supplementation with selective bacteria holds promise in positively affecting the endocrine system. In mice, the microbiota can regulate testicular development and function [251], while androgen deficiency has substantially altered the microbiome [252]. Supplementation with a selenium-enriched probiotic in conjunction with a high-fat diet in male mice significantly alleviated the adverse effects of hyperlipidemia by reducing testicular tissue injury, increasing serum testosterone levels, and improving sperm indexes [253]. Further, aging mice supplemented with the probiotic bacterium *L. reuteri* had larger testicles and increased serum testosterone levels compared to their age-matched controls [237, 254].

In a human pilot study, supplementation with *L. acidophilus* and *B. longum* (1 × 10^9^ CFU) did not alter plasma hormones, including testosterone, dihydrotestosterone, androstenedione, dehydroepiandrosterone sulfate, and sex hormone-binding globulin, in 31 healthy males (18 to 37 years old) over a two-month period [255]. However, another pilot study supplementing a probiotic and prebiotic (*L. paracasei* B21060 5 × 10^9^ cells + arabinogalactan 1243 mg + fructooligosaccharides 700 mg + L-glutamine 500 mg) over 6 months in infertile male patients improved gonadal pathway function including increased follicle stimulating hormone, luteinizing hormone, and testosterone levels compared to a control group [256].

Interestingly, Tremellen et al. [257] proposed that gut-derived endotoxin can reduce gonadal function in obese males. Obesity and a high fat/high calorie diet can alter gut bacteria and intestinal wall permeability, leading to the passage of LPS from within the gut lumen into the circulation (metabolic endotoxemia), where it initiates systemic inflammation [258]. Endotoxin can reduce testosterone production by the testes, both by direct inhibition of Leydig cell steroidogenic pathways and indirectly by reducing pituitary luteinizing hormone drive and sperm production [259]. Tremellen and colleagues [257] theorized the male reproductive axis has evolved the capacity to lower testosterone production during times of infection and resulting endotoxin exposure, decreasing the immunosuppressive influence of testosterone, in turn enhancing the ability to fight infection. Weight loss and physical activity seem to improve these symptoms [260]. These novel findings suggest a potential impact for microbe therapy in obese and/or aging athletes by imparting hormonal and gonadal features of reproductive fitness typical of much younger healthy individuals. However, studies are severely lacking. In the future, larger sample sizes and more robust study designs will be needed.

#### Inactivated “probiotics”

There is an increasing interest in supplementation with non-viable microorganisms or microbial cell extracts. By definition, probiotics are required to be alive, therefore inactivated microorganisms cannot be classified as such. However, preparations from certain probiotic species and strains (such as those from lactobacilli and bifidobacteria) have shown to maintain health benefits even after no longer being viable [261–263]. Inactivation can be achieved by different methods, including heat, chemicals (e.g., formalin), gamma or ultraviolet rays, and sonication, with heat treatment being the method of choice in most cases [228, 264, 265]. Importantly, these methods of inactivation may affect structural components of the cell differently, and therefore their biological activities [264, 265]. Piqué et al. (2019) suggested the presence of key structures in the cell or supernatant fractions may confer probiotic properties, mainly through immune-modulation, protection against pathogens, and fortifying the mucosal barrier integrity [261]. These different bacterial components include lipoteichoic acids, peptidoglycans, and/or exopolysaccharides [261].

Favorable properties of heat-killed bacteria have been observed in vitro [266], in animal models [264], and human trials [267, 268]. For example, in healthy subjects with high levels of self-reported psychological stress, supplementation with heat-killed *L. plantarum* L-137 significantly lowered incidence of URTI after 12 weeks compared to the control group [269]. This finding may have resulted from innate immunity stimulation as heat-killed *L. plantarum* L-137 has been reported to enhance type I IFN production in humans [270]. In athletes, there have only been two studies published examining the effect of these inactivated “probiotics”. In a randomized, double blind, placebo-controlled trial, 51 male athletes engaged in high intensity exercise (> 11 h per week) and consumed a placebo or heat-killed *L. lactis* JCM 5805 daily for 13 days [262]. Compared to placebo, supplementation increased the maturation marker of plasmacytoid DC pDC (CD86), responsible for the antiviral response, and decreased the cumulative days of URTI symptoms. Furthermore, ingestion decreased cumulative days of self-reported fatigue. In a longer duration randomized, double blind, placebo-controlled study, 49 long-distance runners consumed heat-inactivated *L. gasseri* CP2305 or placebo daily for 12 weeks [271]. No significant difference in physical performance between the CP2305 and placebo group were detected. However, CP2305 supplementation improved recovery from fatigue and relieved anxiety and depressive mood compared with placebo intake. Further, CP2305 intake prevented training-induced reduction of hemoglobin and facilitated exercise-induced increase in serum growth hormone levels. Moreover, gene expression profiling of peripheral blood leukocytes indicated that CP2305 prevented the stress-induced changes in the expression of genes related to mitochondrial functions. In relation to the gut microbiota, CP2305 intake increased the alpha- and beta-diversity, and the compositions of *Bifidobacterium* and *Faecalibacterium*. These compositional changes in the gut microbiota may have contributed to the recovery of fatigue and moderation of stress and anxiety through the gut-brain axis. Indeed, inactivated CP2305 can relieve stress in healthy young adults facing stressful conditions [272]. While encouraging, it is unclear how the daily intake of the heat-inactivated probiotics could affect the gut-brain axis and alter stress responses. Further research investigating potential mechanisms as well as more extensive studies with a wider range of athletes and exercise loads should be conducted. In addition, primary aims related to GI tract health and exercise performance should be more thoroughly assessed.

#### Mood and cognition

Physical health and mental health are strongly linked with depression, which is recognized as a leading cause of disability throughout the world [273]. Recently, it has been reported that 35% of individuals with depression also have symptoms of a leaky gut [274], which strengthens the notion of a link between the brain and the GI tract. As reported by Clarke et al. [275], gut bacteria contribute to various mood states in an individual. The gut-brain axis is a bidirectional pathway via the neural, endocrine, and immune systems. The mechanisms by which probiotics improve symptoms of depression and other mood disorders are via anti-inflammatory actions that reduce activity of the hypothalamic-pituitary-adrenal (HPA) axis [276].

Probiotics may be an effective treatment strategy for depression and mood disorders such as anxiety given the link between GI tract bacteria and the brain (i.e. the gut-brain axis), as decreased intestinal dysbiosis may have beneficial effects on mood. Only a few studies have been completed in human subjects that have examined the impact of probiotic supplementation on mood and anxiety. Benton and colleagues [210] reported that 3 weeks of supplementation with 1 × 10^8^ CFU of *L. casei* had positive effects on mood, with subjects feeling increased clear-headedness, confidence, and elation compared to baseline. A study by Rao et al. [277], reported that 8 weeks of 8 × 10^7^ CFU of *L. casei* given to individuals with chronic fatigue syndrome reduced anxiety symptoms. Similarly, Messaoudi and others [278] found decreased anxiety related behaviors after 2 weeks of a combination of *L. helveticus* and *B. longum* in 25 healthy adults. Moreover, 6 weeks supplementation of 4 × 10^9^ CFU/live cells of *L. fermementum* LF16, *L. rhamnosus* LR06, *L. plantarum* LP01, and *B. longum* BL04 improved mood and sleep quality with a reduction in depressive mood state, anger and fatigue [279].

Overall, research on probiotics and mood in athletic populations is lacking. One review, completed by Clark and Mach [280] likened the psychological demands of exercise to physical stress. These authors concluded that the gut microbiota acts as an endocrine organ, secreting neurotransmitters such as serotonin and dopamine, thereby controlling the hypothalamic-pituitary axis in athletes. It is unclear whether these conclusions are attributable to the physiological or psychological stress, and more research is needed to expand on the current findings.

#### Muscle damage and recovery

Inflammation has been implicated in probiotic supplementation impacting body fat levels in overweight and obese individuals, as well as athletic populations. Research in this area, however, has been completed entirely in animal models. Zhao et al. [281] reported that supplementation of *Akkermancia muciniphila* in lean mice fed a chow diet for 5 weeks significantly improved markers of low-grade, chronic inflammation via measurement of LPS, and alleviated gains in both body weight and fat mass. Probiotic supplementation also increased anti-inflammatory factors α-tocopherol and β-sitosterol. Interaction between *A. muciniphila* and inflammatory processes may subsequently impact metabolic health and consequently body composition regulation. In humans, low-grade, chronic inflammation is a marker of many disease states and aspects of the metabolic syndrome. To date, no such research has been completed in athletic populations to clarify the impact of probiotic supplementation on body composition in athletes.

#### Neurotransmitter synthesis and release

Choline and its derivatives serve as components of structural lipoproteins, blood and membrane lipids, and as a precursor of the neurotransmitter, acetylcholine [282]. Choline is converted into acetylcholine via the enzyme choline acetyltransferase. Increasing plasma levels of choline could improve the production of acetylcholine, increase muscular contraction, and possibly delay fatigue in endurance exercise [282]. Elevated choline levels were observed in plasma of mice supplemented with *L. rhamnosus* compared to those fed with *L. paracasei* and controls [283]. In humans, probiotics and choline have been studied in the context of Trimethylamine N-oxide (TMAO). TMAO is an atherogenic metabolite that requires gut microbes for its generation through a metaorganismal pathway that begins with dietary consumption of trimethylamine (TMA) containing precursors such as choline, carnitine and phosphatidylcholine [284]. In a two-week clinical study on 19 healthy, non-obese males, supplementing with a multi-strain probiotic following a hypercaloric, high-fat diet failed to elevate plasma choline levels [285]. In a three-month pilot study investigating the effects of probiotic supplementation on TMAO plasma levels in hemodialysis patients, choline did not change compared to control group [286]. There is currently no research in athletes or active individuals, yet increases in plasma choline could (in theory) support increases in acetylcholine and consequently power, and endurance.

#### Nutrient timing

As indicated previously, various supplementation protocols have been implemented regarding probiotic consumption supplementation, including taking on an empty stomach, with food, and even after exercise. In relation, little is known pertaining to the optimal timing of probiotic intake for improved microbial survival and nutrient absorption. Tompkins et al. utilized an in vitro digestive model of the upper GI tract to investigate the timing effects of probiotic intake utilizing a multi-species encapsulated product containing *L. helveticus* R0052, *L. rhamnosus* R0011, *B. longum* R0175, and *S. cerevisiae boulardii* [287]. Results of this investigation showed that when a probiotic was consumed 30 min before a meal or with a meal, the bacteria survived in high numbers. Conversely, when the probiotic was taken 30 min after a meal, the bacteria did not survive in high numbers. Additionally, this study reported that consumption of the probiotic with 1% milk and oatmeal-milk gruel allowed for higher bacteria survival than when consumed with apple juice or spring water. Thus, future work should focus on the most favorable time to consume probiotics to promote survival in humans along with optimal nutrient/foodstuffs co-ingestion.

#### Response to a physical or mental stressor

Cortisol is a steroid hormone released by the adrenal glands in response to stress and increased levels have been related to suppression of the immune system in athletes [288–290]. Moreover, a connection has been established between the digestive tract and stress [291, 292]. Several studies that supplemented healthy young college students during exam preparation with probiotics (*L. plantarum* 299v and *L. casei* Shirota) reported attenuation of cortisol compared to a control group [293–295]. However, in an eight-week crossover design, 29 healthy male volunteers who supplemented with *L. rhamnosus* exhibited little difference in stress-related measures, HPA axis response, inflammation, or cognitive performance in comparison to placebo [296]. More recently, a systematic review and meta-analysis of clinical and pre-clinical literature on the effects of probiotics on anxiety asserted that probiotics may help reduce anxiety [297]. However, these findings have not yet been fully translated in clinical research in humans. More relevant to performance, eight endurance-trained males in a blinded randomized crossover design who supplemented with a probiotic beverage (*L. casei*, 1 × 10^11^ CFU) for seven consecutive days before a two-hour running exercise at 60% VO_2_max in hot ambient conditions (34.0 °C and 32% RH) failed to exhibit a decrease in cortisol response compared to a placebo [75].

**Table:** 

**Key Points 8 – Future Directions**	
• Probiotic therapy has the potential to positively affect the endocrine system (testosterone production), especially for obese and/or aging athletes.	
• Modulation of the gut microbiome could alter the production/level of important neurotransmitters related to athletic performance.	
• Probiotic supplementation may have an impact on stress; however, current research is limited.	
• Preliminary animal research suggests probiotic supplementation may support the removal and utilization of blood lactate.	
• Important methodological considerations must be addressed systematically in future research including the effect of: sex, sample size, duration, dose (type and amount), level of physical activity, and type of exercise.	

## Summary

Understanding whether probiotic supplementation plays a role in athletic performance is of interest to athletes who work to improve their training and competition performance. Moreover, this knowledge may be of general benefit to human health. Further studies are required to understand how the microbiome influences anti-inflammatory effects, optimal breakdown and utilization of consumed food, and other beneficial effects for overall health in athletes. Overall, the studies reviewed in this position statement provide modest evidence that probiotics can provide some clinical benefits in athletes and other highly active individuals (Table 3). The difficulty in interpreting the studies is illustrated by variations in clinical outcome measures and most importantly, as probiotic benefits are strain-specific, by different strains used in these studies.

As outlined in Table 3, the following probiotic strains/species have been linked to an increase in athletic performance and/or recovery:

1. *B. coagulans* GBI-30, 6086 (BC30) at 1 × 10^9^ CFU has beneficial effects in combination with protein on exercise recovery;
2. Encapsulated *B. breve* BR03 in combination with *S. thermophilus* FP4 at 5 × 10^9^ CFU each has beneficial effects on exercise recovery and performance following muscle-damaging exercise;
3. *L. delbrueckii* ssp. *bulgaricus* at 1 × 10^5^ CFU can increase VO_2_max and aerobic power;
4. *L. acidophilus* SPP, *L. delbrueckii bulgaricus*, *B. bifidum*, and *S. salivarus thermophilus* at 4 × 10^10^ CFU administered in form of a yogurt drink can increase VO_2_max;
5. *L. plantarum* TWK10 at 1 × 10^10^ CFU has been shown to increase endurance performance;
6. *L. acidophilus*, *L. rhamnosus*, *L. casei*, *L. plantarum*, *L. fermentum*, *B. lactis*, *B. breve*, *B. bifidum* and *S. thermophilus* at 4.5 × 10^10^ CFU can increase run time to fatigue in the heat.

The following probiotic strains/species have been linked to improved gut health in athletes (see Table 3):

1. *L. rhamnosus* GG at 4 × 10^10^ CFU in form of a milk-based drink,
2. *B. bifidum* W23, *B. lactis* W51, *E. faecium* W54, *L. acidophilus* W22, *L. brevis* W63, and *L. lactis* W58, at 1 × 10^10^ CFU;
3. *L*. *salivarius* (UCC118) (unknown dose).

The following strains/species have been shown to improve immune health in athletes, reducing the episodes, severity or duration of exercise-induced infections:

1. 1.2 × 10^10^ CFU *L. fermentum* VRI-003 (PCC) at 1.2 × 10^10^ CFU and at 1 × 10^9^ CFU in males;
2. *L. casei* Shirota (LcS) at 6.5 × 10^9^ CFU twice daily;
3. *L. delbrueckii bulgaricus*, *B. bifidum*, and *S. salivarus thermophilus* at 4 × 10^10^ CFU administered in the form of a yogurt drink;
4. *B. animalis* subsp. *lactis* BI-04 2 × 10^10^ CFU;
5. *L. gasseri* 2.6 × 10^9^ CFU, *B. bifidum* 0.2 × 10^9^, and *B. longum* 0.2 × 10^9^ CFU;
6. *B. bifidum* W23, *B. lactis* W51, *E. faecium* W54, *L. acidophilus* W22, *L. brevis* W63, *L. lactis* W58 at 1 × 10^10^ CFU;
7. *L. helveticus* Lafti L10 at 2 × 10^10^ CFU.

Given the small number of studies, and substantial variation in experimental approaches, dependent measures, and outcomes, more well-designed studies of probiotic supplementation in various athlete groups are warranted. While a majority of probiotics currently on the market, and tested in humans, feature the *Lactobacillus*, *Bifidobacterium*, and *Bacillus* genera, new microbiome research and technological advances are identifying potential next-generation probiotic candidates. Further research is needed not only to identify these discoveries, and validate their performance and recovery benefits in clinical settings.

## Recommendations

Athletes and physically active individuals should thoroughly review health care and consumer information on specific applications, dosage, and possible contraindications of probiotic supplementation. As with any dietary supplementation, probiotics should be considered in the overall context of balanced dietary intake, i.e. nutrient needs should be met by a “food first” approach via consumption of whole foods rather than supplements. For example, recommending dietary supplements to developing athletes might overemphasize their importance in comparison to other training and dietary strategies [298]. In this context, it is also important to remember that some food-based probiotic products (e.g. yogurt) contain energy, carbohydrate, protein, and other nutrients that can form part of an athlete’s overall nutrition plan. Only reputable sources of commercially available supplements should be used to reduce the risk of contaminants that might contravene doping in sport regulations [5]. Athletes should be educated on the likely risks of contamination given that the World Anti-Doping Agency enforces a principle of strict liability for positive test results involving banned substances. Different formulations of probiotics from tablets or capsules to powder (added to drinks) or probiotic-enriched chewable tablets are available to meet individual preferences.

Probiotic supplements should be packaged, stored, handled, and transported in an appropriate manner. Athletes should take particular care in warm to hot environments and avoid, where possible, leaving supplements outdoors for long periods in direct sunlight, in a motor vehicle, or near an oven or other heat-generating appliances. New technology has led to probiotic supplements that do not require refrigeration, which may be ideal for athletes during travel. Supplements should also be kept dry at all times. During travel it might be useful for individuals to keep probiotics with other nutritional supplies, supplements, ergogenic acids or medications, or held by team personnel as required.

In terms of implementation, probiotic supplementation should commence at least 14 days before a major training period or competition to allow adequate time for transient colonization or adaptation period of bacterial species in the gut. Another important issue is the increased risk of GI problems during travel [299]. Supplementation with probiotics for individuals and athletes traveling could be included in an overall illness prevention plan. Tolerance and side effects should be monitored by the athlete, coach, and support staff and a medical opinion sought if there is ongoing concern. It is not unusual to experience transient increased activity in the gut during the colonization period (e.g., intestinal rumbling, increased flatulence, etc.) and athletes should be informed that mild side effects for a few days are not uncommon [61]. Athletes should be encouraged to review and monitor probiotic consumption on a daily basis to promote compliance and best practice usage. Compliance might be improved by having athletes take the probiotic supplement at the same time each day (e.g., at breakfast). Probiotic supplementation should be tested during the offseason or preseason phases, so the athlete is familiar with taking the probiotic supplements or foods before travel or major competition, and can see how he/she responds. This practice is also useful in the context of assessing individual tolerance and potential adverse effects.

### Position of the International Society of Sports Nutrition (ISSN)

After reviewing the scientific and medical literature in this area, the International Society of Sports Nutrition concludes the following in terms of probiotic supplementation as the official Position of the Society:

1. Probiotics are live microorganisms that, when administered in adequate amounts, confer a health benefit on the host (FAO/WHO).
2. Probiotic administration has been linked to a multitude of health benefits, with gut and immune health being the most researched applications.
3. Despite the existence of shared, core mechanisms for probiotic function, health benefits of probiotics are strain- and dose-dependent.
4. Athletes have varying gut microbiota compositions that appear to reflect the activity level of the host in comparison to sedentary people, with the differences linked primarily to the volume of exercise and amount of protein consumption. Whether differences in gut microbiota composition affect probiotic efficacy is unknown.
5. The main function of the gut is to digest food and absorb nutrients. In athletic populations, certain probiotics strains can increase absorption of key nutrients such as amino acids from protein, and affect the pharmacology and physiological properties of multiple food components.
6. Immune depression in athletes worsens with excessive training load, psychological stress, disturbed sleep, and environmental extremes, all of which can contribute to an increased risk of respiratory tract infections. In certain situations, including exposure to crowds, foreign travel and poor hygiene at home, and training or competition venues, athletes’ exposure to pathogens may be elevated leading to increased rates of infections. Approximately 70% of the immune system is located in the gut and probiotic supplementation has been shown to promote a healthy immune response. In an athletic population, specific probiotic strains can reduce the number of episodes, severity and duration of upper respiratory tract infections.
7. Intense, prolonged exercise, especially in the heat, has been shown to increase gut permeability which potentially can result in systemic toxemia. Specific probiotic strains can improve the integrity of the gut-barrier function in athletes.
8. Administration of selected anti-inflammatory probiotic strains have been linked to improved recovery from muscle-damaging exercise.
9. The minimal effective dose and method of administration (potency per serving, single vs. split dose, delivery form) of a specific probiotic strain depends on validation studies for this particular strain. Products that contain probiotics must include the genus, species, and strain of each live microorganism on its label as well as the total estimated quantity of each probiotic strain at the end of the product’s shelf life, as measured by colony forming units (CFU) or live cells.
10. Preclinical and early human research has shown potential probiotic benefits relevant to an athletic population that include improved body composition and lean body mass, normalizing age-related declines in testosterone levels, reductions in cortisol levels indicating improved responses to a physical or mental stressor, reduction of exercise-induced lactate, and increased neurotransmitter synthesis, cognition and mood. However, these potential benefits require validation in more rigorous human studies and in an athletic population.

## Conclusion

Given all the known benefits and favorable safety profile of probiotic supplementation reported in the scientific and medical literature, probiotics are commonly used to optimize the health of athletes. Regular consumption of specific probiotic strains may assist with immune function and may reduce the number of sick days an athlete experiences when training or during competition. Certain probiotic strains may reduce the severity of respiratory infection and GI disturbance when they occur. Probiotic benefits are strain specific and dose dependent, and include improved gut-barrier function, nutrient absorption, recovery and performance in athletes. When choosing a probiotic product, athletes are encouraged to use clinically researched strains with validated benefits, matching the athletes desired health benefit. Studies investigating the effects of probiotics in athletic populations and on sports performance are limited and warrant further investigation.

## Data Availability

Not applicable.

## Abbreviations

AE: Adverse events
ANVISA: National Health Surveillance Agency Brazil
ATP: Adenosine triphosphate
BCAAs: Branched-chain amino acids
BMI: Body mass index
BUN: Blood urea nitrogen
CD14: Cluster of differentiation factor-14
CFU: Colony forming units
CLA: Conjugated linoleic acid
CLR: C-type lectin receptor
CRN: Council for Responsible Nutrition
CRP: C-Reactive protein
EFSA: European Food Safety Authority
FAO: Food and Agricultural Organization
FDA: Food and Drug Administration
FOSHU: Foods for Specific Health Uses
GI: Gastrointestinal
GPR: G-Protein couple receptor
HIV: Human immunodeficiency virus
HPA: Hypothalamic-pituitary-adrenal axis
IBD: Inflammatory bowel disease
ICR: Institute of Cancer Research
IgA: Immunoglobulin A
IL-1β: Interleukin-1beta
IL-6: Interleukin-6
IOC: International Olympic Committee
IPA: International Probiotic Association
ISSN: International Society of Sports Nutrition
LPS: Lipopolysaccharide
MHLW: Ministry of Health and Welfare
NK-κβ: Nuclear factor kappa beta
NOD: Nucleotide-binding oligomerization domain
PAG: Phenylacetylglutamine
PAMP: Pathogen associated molecular pattern
PCR: Polymerase chain reaction
PPR: Pattern recognition receptors
RNA Seq: RNA sequencing
SFCA: Short chain fatty acid
TLR: Toll-like receptor
TMAO: Trimethylamine N-oxide
TNF-α: Tumor necrosis factor-alpha
Treg: Regulatory T cells
URTI: Upper respiratory tract infection
VO_2_: Volume of oxygen utilization
WGO: World Gastroenterology Organization
WHO: World Health Organization
